# Trait coordination and environmental filters shape functional trait distributions of forest understory herbs

**DOI:** 10.1002/ece3.7000

**Published:** 2020-11-26

**Authors:** Matt Candeias, Jennifer Fraterrigo

**Affiliations:** ^1^ Department of Natural Resources and Environmental Sciences University of Illinois Urbana IL USA; ^2^ Program in Ecology, Evolution, and Conservation Biology University of Illinois Urbana IL USA

**Keywords:** community assembly, environmental filtering, environmental gradient, leaf economics spectrum, plant traits, trait coordination

## Abstract

1. Understanding the drivers of trait selection is critical for resolving community assembly processes. Here, we test the importance of environmental filtering and trait covariance for structuring the functional traits of understory herbaceous communities distributed along a natural environmental resource gradient that varied in soil moisture, temperature, and nitrogen availability, produced by different topographic positions in the southern Appalachian Mountains.
2. To uncover potential differences in community‐level trait responses to the resource gradient, we quantified the averages and variances of both abundance‐weighted and unweighted values for six functional traits (vegetative height, leaf area, specific leaf area, leaf dry matter content, leaf nitrogen, and leaf δ^13^C) using 15 individuals of each of the 108 species of understory herbs found at two sites in the southern Appalachians of western North Carolina, USA.
3. Environmental variables were better predictors of weighted than unweighted community‐level average trait values for all but height and leaf N, indicating strong environmental filtering of plant abundance. Community‐level variance patterns also showed increased convergence of abundance‐weighted traits as resource limitation became more severe.
4. Functional trait covariance patterns based on weighted averages were uniform across the gradient, whereas coordination based on unweighted averages was inconsistent and varied with environmental context. In line with these results, structural equation modeling revealed that unweighted community‐average traits responded directly to local environmental variation, whereas weighted community‐average traits responded indirectly to local environmental variation through trait coordination.
5. Our finding that trait coordination is more important for explaining the distribution of weighted than unweighted average trait values along the gradient indicates that environmental filtering acts on multiple traits simultaneously, with abundant species possessing more favorable combinations of traits for maximizing fitness in a given environment.

## Graphical abstract

Understanding the drivers of trait selection is critical for resolving community assembly processes. Our finding that trait coordination is more important for explaining the distribution of weighted than unweighted average trait values along the gradient indicates that environmental filtering acts on multiple traits simultaneously, with abundant species possessing more favorable combinations of traits for maximizing fitness in a given environment.

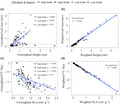

## 1 INTRODUCTION

Understanding the factors influencing plant community assembly is central to explaining the formation and maintenance of biodiversity (Rosindell et al., 2011) and how communities respond to environmental change (Díaz et al., 2004; Shipley et al., 2006). Functional trait‐based approaches offer valuable insights into the community assembly process by providing a mechanistic link between plant traits and the environment while avoiding the idiosyncrasies associated with site‐specific patterns of taxonomic community composition (Jiang et al., 2018; Siefert et al., 2013). Functional traits are heritable attributes that reflect specific physiological and morphological adaptations to abiotic and biotic constraints, thus indicating the diverse ecological strategies plants use to survive and co‐exist under differing environmental conditions (Westoby, 1998; Westoby & Wright, 2006). Identifying the drivers of trait selection along environmental gradients at local scales is particularly important for supporting conservation and management actions.

Multiple mechanisms have been proposed to explain the processes of plant community assembly. These mechanisms generally fall into two categories: environmental filtering, which is expected to increase similarities among plant traits based on the degree of trait–environment correspondence (Cornwell et al., 2006; Weiher & Keddy, 1995), and competitive interactions (i.e., niche partitioning, limiting similarity), which are expected to prevent coexistence among species with similar trait values (Chesson, 2000; MacArthur & Levins, 1967). Broad‐scale analyses of plant functional traits have revealed mixed support for both categories, indicating that multiple mechanisms operate simultaneously during the community assembly process (Cahill et al., 2008; Mayfield & Levine, 2010; Spasojevic & Suding, 2012). How these mechanisms operate on individual and multivariate traits is likely to have important consequences for species presence–absence and abundance within a community (Cingolani et al., 2007).

Plant community responses can involve both changes in species presence–absence and abundance. Community‐level trait averages are generally weighted by species relative abundances, and are thus expected to reveal trait values that maximize fitness and performance under a given suite of environmental conditions (Muscarella & Uriarte, 2016; Shipley et al., 2011). In contrast, community‐level averages based on unweighted trait values for each species at the plot scale reflect changes in species presence–absence. Consequently, weighted and unweighted community‐average trait values may provide complementary information about the community assembly process. Supporting this idea, Cingolani et al. (2007) found different relationships with environmental variables for weighted and unweighted community‐average height: weighted values were positively related to soil moisture whereas unweighted values were unrelated to the measured environmental parameters. This differential response was interpreted as evidence that different factors determine which species will establish upon arriving at a site and which will achieve relative dominance versus remain rare (Cingolani et al., 2007). Evaluating the strength of trait–environment relationships among unweighted and weighted trait distributions may therefore be valuable for determining if multiple environmental filters are indeed influencing plant community assembly.

Traits do not vary independently, with evolutionary and physical trade‐offs leading to multiple functional traits that covary (Reich et al., 1997; Wright et al., 2004). Covariation or coordination among traits reflects the dimensions of trait variation that are hypothesized to maximize fitness within a community. Traits that show little or no coordination with respect to one another are hypothesized to be associated with different ecological strategies (Angert et al., 2009; Dwyer & Laughlin, 2017b; Wright et al., 2004). For example, among leaf traits, specific leaf area (SLA) is closely correlated with leaf nitrogen (LN), and collectively these traits represent trade‐offs associated with resource acquisition strategies (Wright et al., 2004). In contrast, SLA and plant height (H) are frequently found to vary independently of one another and therefore can provide insights into different ecological strategies such as resource allocation and resource acquisition (Westoby, 1998). Investigating trait coordination in the context of local trait–environment relationships may thus reveal how trade‐offs associated with different ecological strategies influence trait distributions (Dwyer & Laughlin, 2017a).

Trait coordination is expected to strongly constrain community assembly along environmental gradients given that filtering acts on multiple traits simultaneously, thus potentially reducing the number of viable trait combinations present in a community (Dwyer & Laughlin, 2017a; Westoby & Wright, 2006). Yet, how trait coordination affects species abundance versus presence is incompletely understood. If coordinated traits increase fitness, then abundance (as indicated by abundance‐weighted trait values) should be more strongly influenced by trait coordination than presence (as indicated by unweighted trait values) (Funk & Cornwell, 2013; Wright et al., 2001). Trait coordination should also lead to stronger abiotic filtering of weighted than unweighted trait values because maintaining a wide range of trait values across multiple traits simultaneously carries high fitness costs when resource availability is low (Bernard‐Verdier et al., 2012; Dwyer & Laughlin, 2017b). Consequently, weighted trait values may converge as environments become more limiting. In support of this hypothesis, leaf and stem trait coordination in dry tropical forests has been shown to be more convergent compared to leaf and stem trait coordination in wet tropical forests (Baraloto et al., 2010; Markesteijn et al., 2011). Elucidating patterns of trait coordination along environmental gradients and with respect to weighted and unweighted trait values may thus be important for improving understanding of the mechanisms influencing plant community assembly (Dwyer & Laughlin, 2017b; MacLean & Beissinger, 2017).

In this study, we investigated the influence of environmental filtering and trait coordination on the abundance and presence of forest herb species by examining abundance‐weighted and unweighted community‐level trait responses along an environmental resource gradient produced by differences in topographic position (elevation and aspect) in the southern Appalachian Mountains. Changes in elevation and aspect are associated with changes in temperature, moisture, and other environmental factors such as soil nutrient availability over relatively short distances (Körner, 2007). Mountains are thus well suited for examining trait selection in response to environmental variation (Albert et al., 2010; Sundqvist et al., 2011). The novelty of our approach lies in the parallel assessment of community responses to the gradient, with and without consideration of species relative abundances, while accounting for trait coordination. By quantifying leaf and size traits representing different ecological strategies, we addressed three hypotheses. (a) Environmental variables will be stronger predictors of weighted than unweighted plot‐average trait values because environmental filtering on species abundance is stronger than filtering of species presence–absence in a community; (b) Weighted traits will exhibit stronger trait convergence than unweighted traits with increasing resource limitation because species abundance is strongly determined by the magnitude of trait–environment correspondence; (c) Trait coordination will be stronger for and better predict weighted than unweighted plot‐average trait values because abundant species possess combinations of traits that maximize fitness in a given environment.

## 2 METHODS

### 2.1 Study area and experimental design

This study was conducted at two sites in the southern Appalachians of western North Carolina, USA. The Coweeta Hydrologic Laboratory is embedded within the Coweeta basin and located in the Nantahala Mountain Range (35°03′N 83°25′W). The Mainspring Land Trust conservation easement property is located in the Cowee Mountain Range (35°27′N 83°53′W). Regional climate of the southern Appalachians is classified as marine humid temperate with cool summers and mild winters (Swift et al., 1988). The growing season spans early April to October, with the highest temperatures occurring from June to August (~20°C) and the lowest from December to January (~5°C). Mean annual temperature is 12.6°C, and mean annual precipitation is 179 cm. Soils are mostly Inceptisols or Ultisols and are classified as Mesic or Humic Hapludults, or Typic Humudepts. Parent material consists of high‐grade metamorphic rocks (e.g., mica gneiss, mica schist) and metasedimentary rocks (i.e., metasandstone, phyllite, shale) (Block et al., 2012). Mixed deciduous forests with dense perennial understories represent the dominant vegetation communities (Bolstad et al., 1998; Whittaker, 1956). Both sites have a similar history and have not experienced disturbance since they were logged in the early 1900s.

In the spring of 2016, we established twenty 5 m × 5 m study plots in each site (*n* = 40 plots total) along a natural environmental resource gradient produced by different topographic positions. Half of the plots in each site were located on south‐facing slopes and distributed evenly among low‐elevation (850 m) and high‐elevation positions (1,400 m). The other half of the plots were located on north‐facing slopes and distributed in the same manner as described above. Plots in each topographic position were spaced a minimum of 150 m apart to ensure independent observations. We used a Modified‐Whittaker plot design (Stohlgren et al., 1995) in which we randomly nested five 1‐m^2^ subplots within each plot. This design has been shown to enhance the detection and measurement of plant species, especially when vegetation is spatially clustered (Campbell et al., 2002; Fortin et al., 1990; Stohlgren et al., 1995).

### 2.2 Plant traits and environmental data

In June 2016, we recorded the percent cover of all herbaceous species in each subplot. Percent cover was averaged by species across subplots to obtain total cover for each species in each plot. For the most abundant species (representing > 80% of the cumulative cover in each plot) (Pakeman & Quested, 2007), we measured six functional traits that are important for defining the general syndromes of plant resource capture and use (Reich et al., 2003; Westoby, 1998; Wright et al., 2004): vegetative height (H; cm), leaf area (LA; mm^2^), specific leaf area (SLA; mm^‐2^ mg^‐1^), leaf dry matter content (LDMC; mg/g), leaf nitrogen (LN; mg N/g), and leaf δ^13^C (‰). Measurements were taken on three mature individuals of each species from each subplot (*n* = 15 individuals per species per plot) following standardized methods (Pérez‐Harguindeguy et al., 2013). We only sampled from recognizable separate above‐ground individuals, which were assessed by looking for absence of rhizomes or stolons at the base of each plant. Trait functions and measurement details are provided in Appendix S1.

We quantified environmental conditions during the 2016 growing season in each plot. We measured soil temperature continuously (5 cm depth, iButton datalogger), volumetric soil moisture twice per week (7 cm depth, Field Scout TDR 100 probe, Spectrum Technologies), and photosynthetically active radiation (PAR; wavelength: 400–700 nm) twice per month following full canopy leaf out using a 0.5‐m handheld ceptometer (Decagon Devices). To characterize average soil characteristics within each plot, 10 soil cores were collected at random from the upper 10 cm of mineral soil using a 2.2‐cm diameter soil probe. Cores were composited by plot, sieved (<2 mm), and air dried prior to subsampling for determination of pH (1:1 mass: H_2_O volume) and carbon (C) and nitrogen (N) concentrations (Costech 4010 CHNSO Elemental Analyzer, Costech Analytical Technologies). Inorganic nitrogen (N) and phosphorus (P) concentrations were determined by randomly installing three pairs of ion‐exchange resin strips (6.0 cm × 2.5 cm) in the mineral soil at 0–6 cm depth and a minimum distance of 0.5 m from one another in each subplot in June 2016 (Schoenau et al., 1993). Resin strips were left in the ground for 30 days (Coweeta) and 25 days (Mainspring). Upon removal, resin strips were rinsed with DDI water and extracted in 2 mol/L KCl. Extractions were filtered through 0.7‐μm Whatman filter paper and frozen (−20°C) until analysis. We analyzed extracts for NH_4_‐N using the phenolate method, NO_3_‐N using a cadmium column reduction method, and PO_4_ using the molybdenum blue method on a Lachat QuikChem 8500 (Hach Company). All values were expressed on a per day basis and averaged for each plot for use in statistical analyses.

### 2.3 Characterizing the environmental resource gradient

We conducted principal component analysis (PCA) to quantify the leading dimensions of the environmental resource gradient. We used 10 environmental parameters to characterize the contrasting environmental conditions across all plots: average temperature, maximum and minimum temperature, soil pH, total soil N concentration, NH_4_‐N, NO_3_‐N, PO_4_, average soil moisture, and PAR. We performed PCA on the correlation matrix after scaling and centering the environmental parameters. We used analysis of variance (ANOVA) to assess differences in average environmental variables across each topographic position (high elevation, north‐facing slope: HN; high‐elevation, south‐facing slope: HS; low‐elevation, north‐facing slope: LN; low‐elevation, south‐facing slope: LS).

### 2.4 Relating unweighted and weighted trait values to the resource gradient

For each trait in each plot, we calculated unweighted and weighted trait averages as:

(1) $$\text{Total}\;\text{unweighted}\;\text{trait}\;\text{average}=x_{\mathrm{ij}}$$

(2) $$\text{Total}\;\text{weighted}\;\text{trait}\;{\text{average}}_{j}=\sum_{i=1}^{S}\mathrm{pijxj}$$

where *p_ij_* is the relative cover of species *i* in plot *j*, *x_ij_* is the mean trait value of species *i* measured in plot *j*, and *S* is the number of species sampled in the plot.

To test for environmental filtering among individual unweighted and weighted plot‐average trait values across the resource gradient, we used linear mixed effect models and an information theoretic approach. We first developed a set of candidate models including the plot scores of the first two PCA axes, environmental variables, elevation, aspect, and canopy cover as fixed effects and site as a random effect. Because we measured functional traits that are explicitly linked to species composition, relating CWM trait values to environmental variables via linear mixed effect models does not run the risk of inflated Type I errors and therefore is fully justified (Zelený, 2018). For each of the candidate models, we included highly correlated predictor variables (soil N:soil C, |*r*| > .6) and PCA axes scores in separate models to reduce collinearity and the number of variables in model. Variables were log‐transformed as needed to meet assumptions of normality and equal variance. Support for models was assessed using the Akaike information criterion corrected for small sample sized (AICc). We considered models to have competitive support when AICc weights were >0.95. Because the number of fixed effects varied between models, maximum likelihood estimation was used for model selection. Parameter estimates for the final model were calculated using restricted maximum likelihood estimation. We considered variables with *p* < .05 as significant and *p* < .10 as marginally significant (sensu Hurlbert & Lombardi, 2009). Analyses were performed using the AICcmodavg package in R (Mazerolle, 2016).

To evaluate the relative contributions of interspecific (variation in trait values among species) and intraspecific (variation in trait values within species) trait variation to total among‐plot variation of unweighted and weighted average trait values along the resource gradient, we partitioned trait variance components using the approach outlined by Lepš et al. (2011). Using the unweighted and weighted plot‐average trait values, we computed three types of trait averages: (a) total trait average, reflecting the effect of both species turnover and ITV, (b) interspecific trait average, and (c) intraspecific trait average. Calculations were performed as follows:

Total unweighted and weighted trait averages were calculated as outlined in Equations 1 and 2.

(3) $$\text{Intraspecific}\;\text{trait}\;{\text{average}}_{j}=\text{Total}\;\text{trait}\;{\text{average}}_{j}‐\text{Interspecific}\;\text{trait}\;{\text{average}}_{j}$$

We tested for differences in the relative contributions of inter‐ and intraspecific trait variability between unweighted and weighted averages across all traits using paired *t* tests.

### 2.5 Quantifying trait dispersion

To test for differences in the dispersion of single, unweighted plot‐average traits across the environmental resource gradient, we used Levene's test with unweighted traits as dependent variables and elevation and aspect as independent variables (Levene, 1960). For single weighted trait values, we calculated community‐weighted variance (CWV) for each trait *t* in each plot, with each species, *i*, weighted by its relative abundance, *p_i_* (Bernard‐Verdier et al., 2012):

(4) $$\text{CWV}=\sum_{i=1}^{n}p_{i}\times {\left(t_{i}‐\text{CWM}\right)}^{2}$$

We then estimated the extent to which community trait variance differed from what is expected from a random null model (i.e., an “effect size”). We compared our observed values of CWV for each trait across gradients with a random distribution generated by 9,999 runs of a null model. The null model shuffles species abundance values randomly while the list of observed species and their associated traits in each community remains unchanged. Randomization breaks any relationship between trait values and abundances, while maintaining the same richness and evenness of abundances in each plot, allowing the examination of single‐trait dispersion patterns for abundance‐weighted traits in response to environmental variation. We calculated an effect size (ES) based on the probability of each observed value being lower than expected by chance (i.e., the quantile of the null distribution in which the observed value is found) (Bernard‐Verdier et al., 2012; Chase & Myers, 2011). We rescaled ES values to vary from −1 to 1 and interpreted negative values as an indication of a lower observed dispersion than expected and vice versa (Bernard‐Verdier et al., 2012). To evaluate whether there was a significant departure in observed ES from the null expectation (trait convergence or trait divergence) across the entire community, we used a two‐tailed Wilcoxon signed‐ranks test (*W*) on ES values to test if they were overall different from zero (positive or negative) (Sokal & Rohlf, 1995). To test for significant trends between dispersion patterns, the environmental resource gradient, and topographic position, and because ES data were nonparametric, we used nonparametric Spearman's rank correlation analysis (S) (Bernard‐Verdier et al., 2012).

### 2.6 Testing for changes in unweighted and weighted trait covariation

To investigate how patterns of unweighted and weighted trait covariation change across the environmental resource gradient, we used a modified permutation test to generate a two‐tailed significance value for trait covariation at the unweighted and weighted levels at each topographic position. Permutation tests correct for inflated Type I error rate, especially when relating weighted trait values (Zelený, 2018; Zelený & Schaffers, 2012). Specifically, for each trait combination, we generated 9,999 permutations of individual unweighted and weighted trait values for one trait while keeping the original values of the other trait intact. The permuted trait values represent the null hypothesis of no covariation among traits. We tested for correlation among measured trait combinations at unweighted and weighted levels against the 9,999 randomized samples using nonparametric Spearman's Rank correlation analysis (*S*). Unweighted and weighted trait correlations were considered significant if the correlation coefficient of measured trait values fell within the lower or upper 95th percentile of the randomized null distribution. Permutation tests were performed using the wPerm package in R (Weiss, 2015).

### 2.7 Estimating the direct and indirect effects of environmental variables and trait covariation on unweighted and weighted trait distributions

To determine the relative roles of environmental variables and trait covariation for predicting individual trait distributions, we used structural equation modeling. Our specific goal was to test the prediction that unweighted trait averages would be structured largely by environmental variables whereas weighted trait averages would be structured largely by trait coordination. Our approach was to generate structural equation models (SEMs) that were consistent with our data with the fewest modifications of the initial model as possible. For this reason, nonsignificant pathways were retained in each model (Grace, 2006). For our conceptual model, trait values are assumed to result from three latent variables: microclimate (climate), soil conditions (edaphic), and leaf trait coordination (leaf economics spectrum). Latent variables are used to define factors that cannot be measured or quantified in their entirety but are hypothesized to be responsible for the outcome of observed measurements. The latent variables were defined using a set of measured variables that serve as indicators of the latent variable (Grace, 2006). The variables that defined microclimate were comprised of soil temp, soil moisture, and PAR, the variables that defined soil conditions were comprised of N availability, P availability, and pH, and the traits that defined leaf trait coordination (LES) were comprised of either unweighted or weighted averages of traits (LA, SLA, LDMC, LN, δ^13^C) identified as significant covariates of the trait of interest by the modified permutation tests described above. Because the modified permutation test corrects for inflated Type I error rate, we were able to avoid biasing our estimates of trait coordination (Zelený, 2018; Zelený & Schaffers, 2012). We fitted the SEMs with the robust maximum likelihood method using the R‐package lavaan (Rosseel, 2012) and constructed the SEMs using the R‐package piecewiseSEM (Lefcheck, 2016). The goodness of fit of each model was evaluated with the chi‐square statistic and the root mean square error of approximation (RMSEA). Chi‐square values higher than 0.05 and RMSEA below 0.05 indicate a good fit between the *SEM* and the observed data (Kline, 2010). The significance of each pathway was evaluated with a *t* test computed on the unstandardized coefficients. To facilitate comparison of effects, we report the standardized path coefficients in the figures. All analyses were conducted in R 3.6.3 (R Core Team, 2018). The data set is publicly available on the EDI Data Portal (https://doi.org/10.6073/pasta/2f2300df5d984581895216fcd96cff08).

## 3 RESULTS

### 3.1 Environmental resource gradient

Across both sites, the most resource‐limited plots were at low elevation, on south‐facing slopes and were characterized by low soil N, low soil moisture, and higher average temperatures. The least resource‐limited plots were at high elevation, on north‐facing slopes and were characterized by high soil N, high soil moisture, and lower average temperatures. PCA identified two significant axes (eigenvalues > 1) of the variation in resource conditions. The first PCA axis (29.4% of the variation explained) was associated with average temperature, maximum temperature, and soil moisture. The second PCA axis (19.7% of the variation explained) was associated primarily with NH_4_‐N availability and PAR (Appendices S3 and S4). On average, high elevation plots were cooler (*p* < .01) and had higher levels of soil nitrogen (*p* < .01 for NO_3_‐N, NH_4_‐N, and total N concentration). Plots on north‐facing slopes had higher levels of soil moisture than plots on south‐facing slopes (*p* = .01), especially at low elevations. Soil pH did not differ significantly among plots (*p* > .05), whereas PAR was lowest in low‐elevation, north‐facing plots (*p* = .03; Table 1).

**TABLE 1:** Environmental conditions (mean ± 1 *SE*) of different topographic positions in western, North Carolina, USA

Environmental variable	Elevation & aspect			
	High North	High South	Low North	Low South
Average temperature (°C)	18.3 ± 0.96^a^	18.7 ± 0.96^a^	19.0 ± 0.96^b^	20.3 ± 0.97^b^
Maximum temperature (°C)	19.6 ± 0.16^a^	20.4 ± 0.19^b^	20.3 ± 0.19^b^	22.1 ± 0.22^c^
Minimum temperature (°C)	16.1 ± 2.41^a^	16.8 ± 2.41^a^	17.0 ± 2.41^a^	18.0 ± 2.42^a^
Soil moisture (%)	15.1 ± 0.56^a^	13.4 ± 0.65^ab^	14.1 ± 0.63^ab^	12.7 ± 0.73^b^
pH	5.03 ± 0.13^a^	4.90 ± 0.14^ab^	5.25 ± 0.14^ab^	5.12 ± 0.15^b^
NO_3_‐N (µg N/cm^2^ day^−1^)	0.45 ± 0.08^a^	0.09 ± 0.08^a^	0.11 ± 0.08^a^	0.05 ± 0.09^b^
NH_4_‐N (µg N/cm^2^ day^−1^)	0.46 ± 0.09^a^	0.40 ± 0.10^ab^	0.27 ± 0.10^ab^	0.10 ± 0.11^b^
PO_4_ (µg P cm^−2^ day^−1^)	0.26 ± 0.13^a^	0.30 ± 0.13^a^	0.15 ± 0.13^a^	0.21 ± 0.14^a^
Soil nitrogen (%)	0.47 ± 0.03^a^	0.36 ± 0.03^a^	0.19 ± 0.03^b^	0.14 ± 0.04^c^
PAR (%)	1.86 ± 0.22^a^	1.42 ± 0.26^ab^	0.85 ± 0.25^ab^	1.30 ± 0.29^b^

Note

Variables were averaged from individual measurements across the two study sites (*n* = 5 for each position). PAR is photosynthetically active radiation (wavelength: 400–700 nm). Superscript letters indicate significant differences.

### 3.2 Trait–environment covariation

We identified a total of 108 herbaceous species across both sites. High elevation plots on north‐facing slopes contained the most species (66), followed by high elevation plots on south‐facing slopes (59), low‐elevation plots on north‐facing slopes (57), and low‐elevation plots on south‐facing slopes (39) (Appendix S2). Interspecific variation contributed significantly more to total among‐plot variation of weighted and unweighted average trait values along the resource gradient (Appendix S6).

**FIGURE 1:**
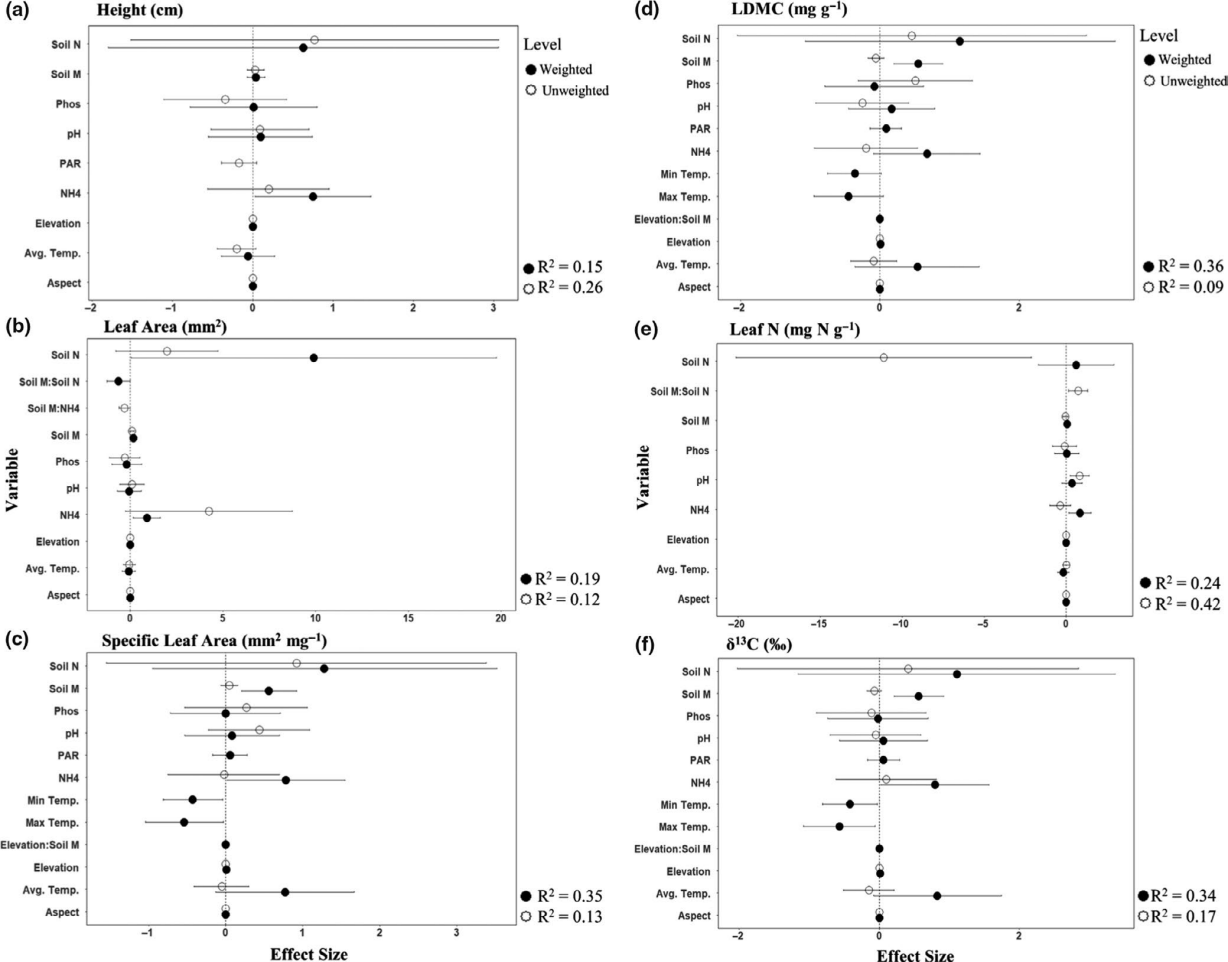
Model coefficient estimates and 95% confidence intervals for predictors included in the confidence set of simple linear regression models explaining weighted (black) and unweighted (white) trait distributions for (a) plant height, (b) Leaf Area, (c) SLA, (d) LDMC, (e) Leaf N, and (f) δ^13^C.*R* ^2^indicates model fit for each level of trait modeling. Continuous predictors were standardized (mean = 0,*SD* = 1) to make effect sizes comparable. Abbreviations are as follows: Avg Temp, average temperature; itmax, maximum temperature; LDMC, leaf dry matter content; Leaf N, leaf nitrogen; Min Temp, minimum temperature; NH_4_, NH_4_‐N; NOx, NO_3_‐N; PAR, photosynthetically active radiation; pH, soil pH; SLA, specific leaf area; Soil M, soil moisture. Full model outputs reported separately in Appendix S7

Unweighted and weighted traits responded similarly to the resource gradient, but environmental variables were stronger predictors of weighted than unweighted plot‐average trait values for all but height (H) and leaf N (LN; Figure 1, Appendix S7). PCA axes scores consistently explained less variation in our trait data than individual environmental variables. Similarly, canopy cover had no significant effect on any of the traits we measured. Unweighted and weighted H, LA, and LN, as well as unweighted SLA, increased significantly with increasing soil moisture (Figure 1a,b,e, Appendix S7). A significant soil moisture × elevation interaction term indicated that weighted SLA and LDMC increased more quickly as soil moisture increased at low elevations (SLA: *β* = 1.70, LDMC: *β* = 1.80) than at high elevations (SLA: *β* = 0.73, LDMC: *β* = 1.10) (Figure 1c,d, Appendix S7). None of the environmental variables explained variation in unweighted LDMC (Figure 1d, Appendix S7). Unweighted δ^13^C became more negative (depleted) as average temperature increased, and a significant soil moisture × elevation interaction term indicated that weighted δ^13^C became depleted more quickly as soil moisture decreased at low elevations (*β* = −1.69) than at high elevations (*β* = −0.68) (Figure 1f, Appendix S7).

### 3.3 Trait dispersion

Plot‐average unweighted traits were under‐dispersed in resource‐limited environments associated with south‐facing slopes and low elevations (Appendix S8). Results of the Levene's Test revealed that the variance of LA (*F*_74_ = 16.4, *p* = <.001) and SLA (*F*_74_ = 6.05, *p* = .01) differed significantly with aspect, with south‐facing slopes exhibiting significantly under‐dispersed trait distributions compared to north‐facing slopes. Aspect had no effect on the variance of unweighted H, LDMC, LN, and δ^13^C (*p* > .05). Unweighted trait variances differed significantly with elevation for H (*F*_74_ = 7.96, *p* = <.001), and marginally so for LA (*F*_74_ = 3.32, *p* = .07), with higher elevations showing larger variances than lower elevations. The variances of LDMC, SLA, LN, and δ^13^C did not differ with elevation (*p* > .05, Appendix S8).

**FIGURE 2:**
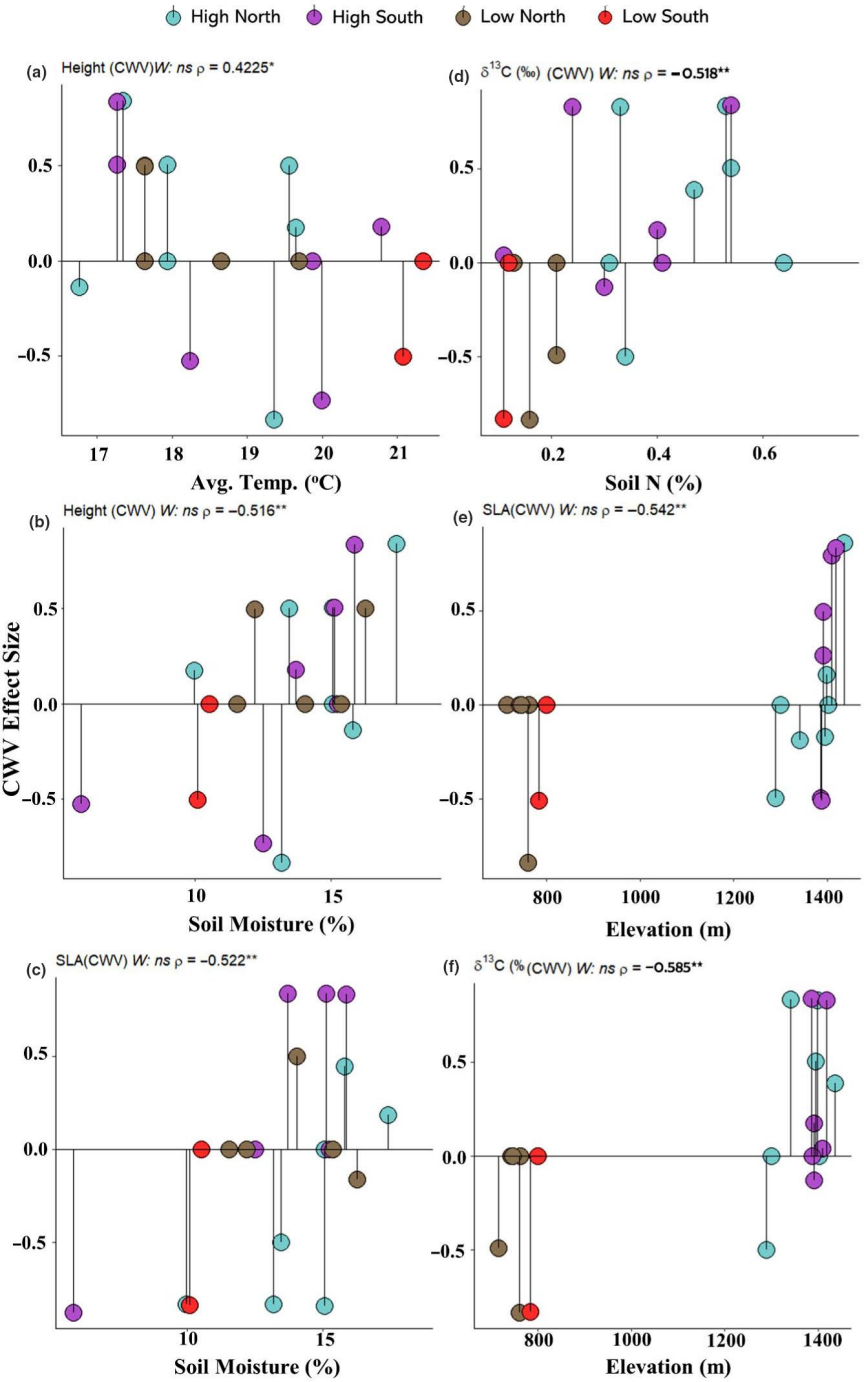
Community‐weighted trait variance (CWV) of height and leaf traits showing (a) CWV height along a temperature gradient, (b) CWV height along a soil moisture gradient, (c) CWV SLA along a soil moisture gradient, (d) CWV δ^13^C along a soil N gradient, (e) CWV SLA along an elevation gradient, and (f) CWV δ^13^C along an elevation gradient. Effect sizes (ES) of CWV were calculated by comparing observed CWV to a null distribution. The solid horizontal line represents the null expectation (ES = 0). Negative ES represents a lower CWV than expected (trait convergence) and positive ES represents a larger CWV than expected (trait divergence). Circles represent landscape position. Statistics for one‐sided Wilcoxon test (*W*) and Spearman's rank correlations (*ρ*) indicated above panels (ns:*p* ≥ .05, *p < .05, **p < .01, ***p < .001)

Abundance‐weighted trait variance patterns were investigated by relating CWV to the expectations of the null model. Considering all plots collectively without abiotic variables, we found no evidence for overall reductions in trait dispersion (nonsignificant Wilcoxon test toward lower values; Figure 2). In contrast, we found evidence of trait under‐ and over‐dispersion in H, SLA, and δ^13^C with respect to environmental resource availability. Limiting environments such as those at low elevation on south‐facing slopes (characterized by high soil temperature, low soil moisture, and low soil N) were associated with lower than expected CWV in H, SLA and δ^13^C, indicating that abundant species tended to converge on similar values under these conditions (Figure 2a–d). Similarly, we found lower than expected CWVs for SLA and δ^13^C in low‐elevation plots (Figure 2e,f).

### 3.4 Trait covariation

**FIGURE 3:**
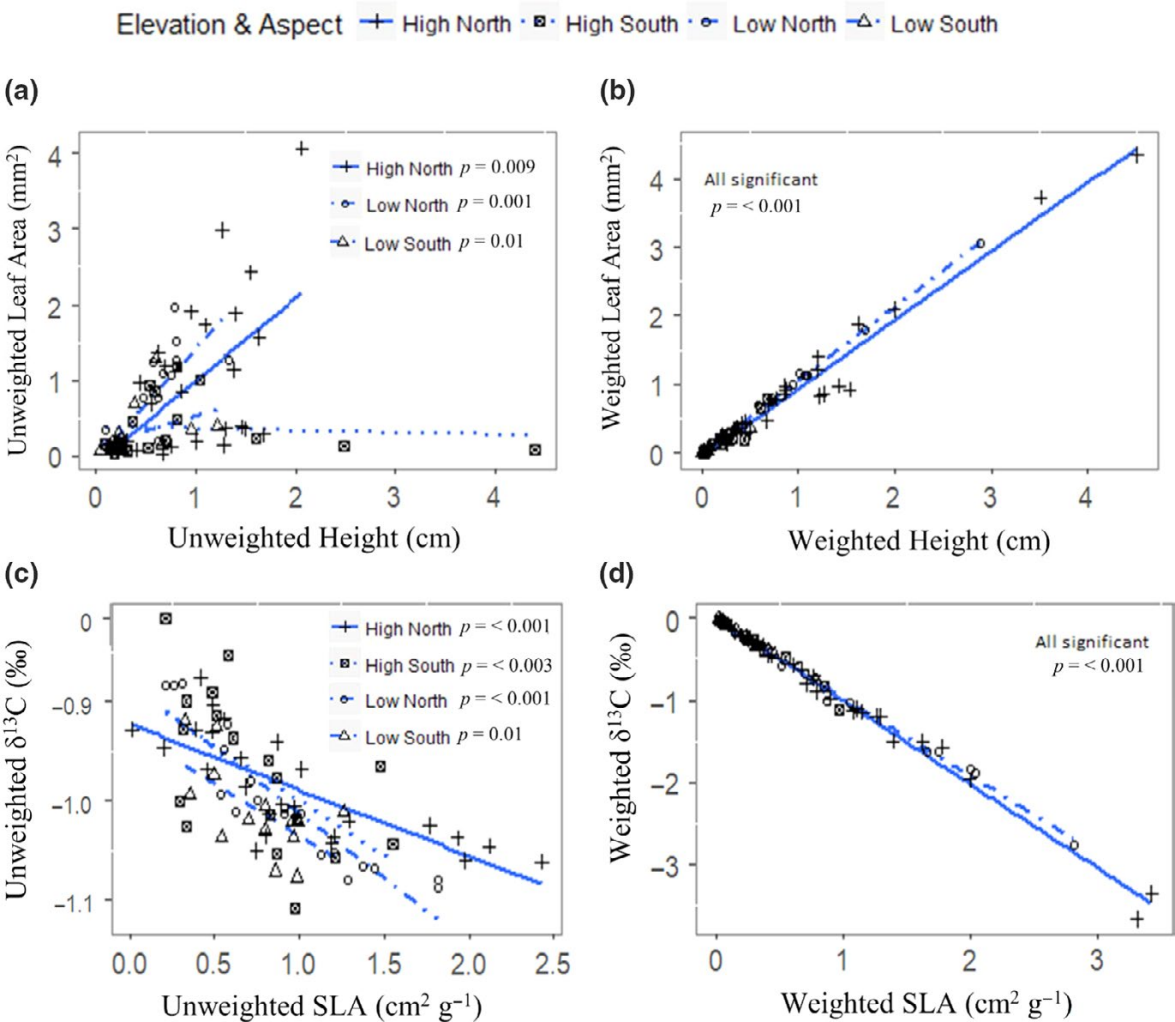
Trait covariation relationships between leaf and height trait averages based on topographic position. Covariation between unweighted height and leaf traits (a) is considerably less than coordination between unweighted leaf traits (c). Unweighted traits (a and c) show context‐dependent coordination whereas weighted traits (b and d) show uniform coordination. Regression lines (blue) are shown for all elevation and aspect combinations and were generated via linear regressions. Significant relationships denoted by*p*‐value within each plot. Axes reflect scaled and centered trait values to enable direct comparisons of effect sizes. See Appendix S10for additional linear relationships

**FIGURE 4:**
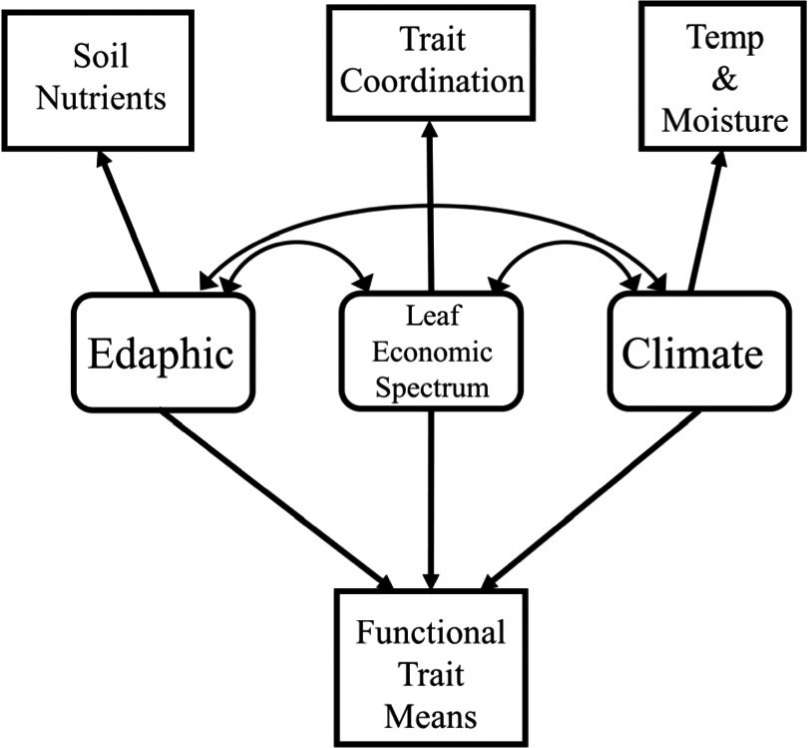
Conceptual model of the direct and indirect influence of edaphic (soil nutrient availability), leaf economics (trait coordination), and climate (temperature and moisture availability) and functional trait averages (unweighted or weighted). Single arrows indicate direct relationships and double arrows indicate the potential for direct and indirect relationships among variables. Squares indicate direct measurements and rounded rectangles indicate latent variables, which are used to define factors that cannot be measured or quantified in their entirety but are hypothesized to be responsible for the outcome of observed measurements

Covariations between leaf traits and plant height at different topographic positions were considerably stronger and more consistent for weighted trait averages than for unweighted trait averages, indicating greater coordination when abundance was considered (Figure 3, Appendix S10). For unweighted trait averages, covariation between leaf traits and plant height differed in strength and direction among topographic positions (Figure 3a,c, Appendix S10a,c,e,g). Significant relationships between unweighted leaf versus height traits were found between H and LA in high‐ (*p* = .009 S = 0.51) and low‐elevation (*p* = <.001, *S* = 0.79), north‐facing plots; low‐elevation, south‐facing plots (*p* = .01, S = 0.64; Figure 3a); and between H and LN in low‐elevation, south‐facing plots (*p* = .03, *S* = 0.56) (Appendix S10a). Overall, leaf traits showed more consistent patterns of covariation than leaf versus height for unweighted trait averages, with leaf traits such as unweighted SLA and δ^13^C significantly related across all topographic positions (Figure 4c) (*p* < .05) except for LN and SLA in low‐elevation, south‐facing plots (Appendix S10g). In contrast, covariations based on weighted trait averages were remarkably consistent among topographic positions, with significant correlations at every topographic position (Figure 3b,d. Appendix S10b,d,f,h).

### 3.5 Direct and indirect effects on trait distributions

**FIGURE 5:**
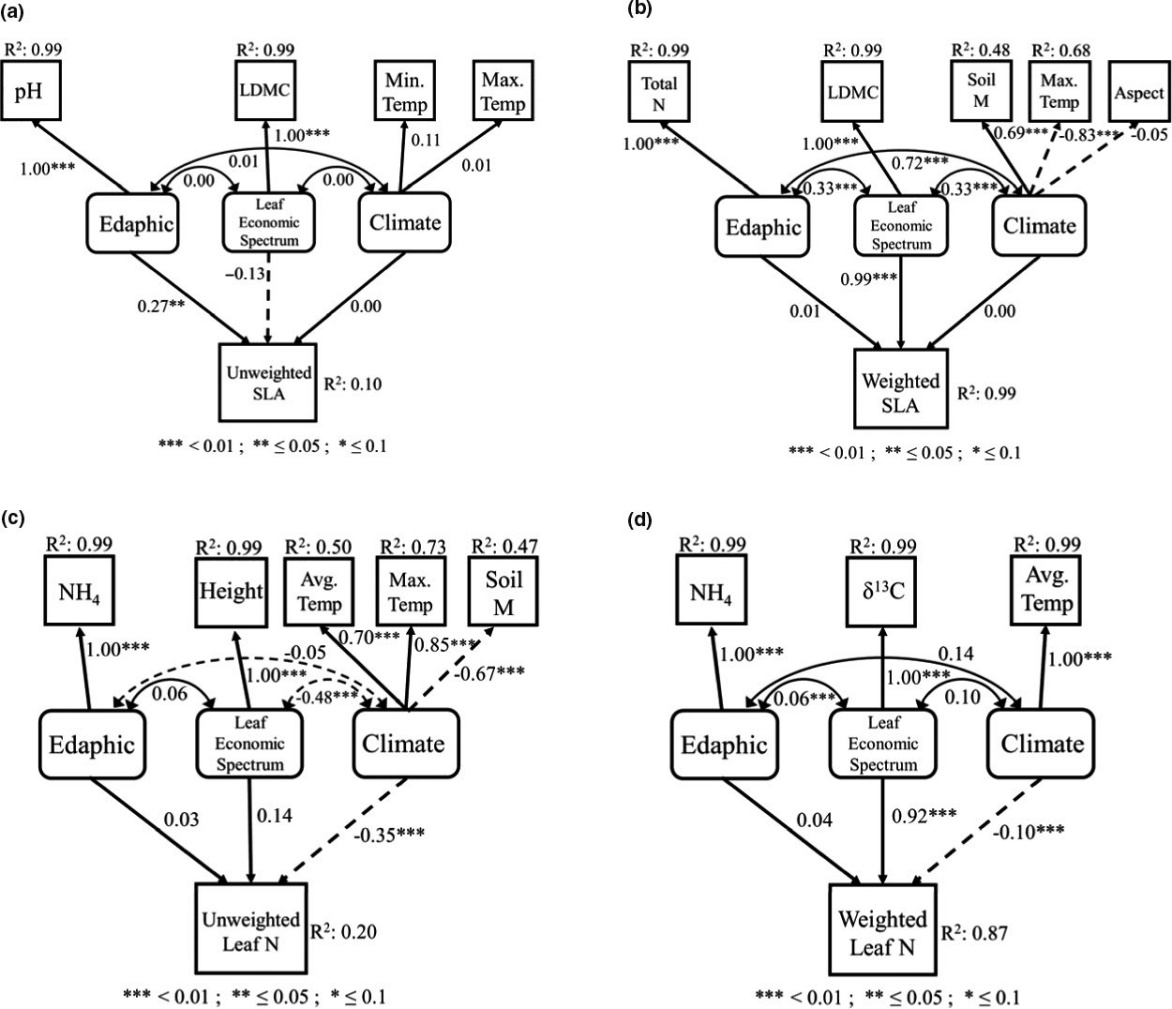
Structural equation models fitted on both unweighted and weighted trait values measured from understory herbs communities in the southern Appalachian Mountains for (a) unweighted SLA, (b) weighted SLA, (c) unweighted LN, and (d) weighted LN. Measured variables are indicated by squares whereas latent variables are indicated by rounded rectangles. Solid arrows indicate positive relationships and dashed arrows indicate negative relationships. Latent variables consist of: Edaphic, soil nutrients; Leaf Economic Spectrum, functional trait averages; and Climate, moisture and temperature. Variable abbreviations are as follows: Leaf area, LA; specific leaf area, SLA; leaf dry matter content, LDMC; leaf nitrogen, Leaf N; maximum temperature, max temp; soil pH, pH; average temperature, avg temp; minimum temperature, min temp; PO_4_, Phos; NH_4_‐N, NH4; PAR; Soil N Concentration, SoilN; soil moisture, soilm; and NO_3_‐N, NOx. Significant paths are indicated by * (***<.01; **≤.05; *≤.1). Double headed arrows refer to covariance estimates.*R* ^2^values show the proportion of variance explained for each variable. We fit models using the robust maximum likelihood method and overall goodness of fit estimated from chi‐square statistic and the root mean square error of approximation (RMSEA). A full list of*SEM*estimates can be found in Appendix S10. For additional SEMs, please see Appendix S11

Structural equation models (Figure 4) demonstrated that environmental variables had strong direct effects on unweighted trait averages, whereas the effects of environmental variables on weighted trait averages were indirect and operated through their influence on trait coordination (Figure 5, Appendices S9 and S11). SEMs specified on unweighted and weighted trait averages adequately fit our data, with all RMSEA values below 0.06 and both CFI and TLI above 0.9 for all models. The exceptions were the models for unweighted and weighted LDMC, which did not show a satisfactory fit. For unweighted SLA, standardized path coefficients indicated a direct effect of soil pH (*R* ^2^ = .99; Figure 5a), whereas weighted SLA trait variation was predicted by indirect contributions of soil N concentration and microclimate (moisture and maximum temperature) variables via trait coordination with LDMC (*R* ^2^ = .99; Figure 5b). Similarly, unweighted LN was explained best by a direct effect of microclimate (average and maximum soil temperature, and soil moisture) (*R* ^2^ = .20; Figure 5c), whereas weighted LN was explained best by indirect contributions from microclimate and soil variables via trait coordination with δ^13^C (*R* ^2^ = 0.92; Figure 5d). Similar patterns occurred in all other unweighted and weighted traits that we measured except for LDMC (Appendices S9 and S11).

## 4 DISCUSSION

Multiple ecological factors can influence plant community assembly and functional trait‐based approaches offer valuable insights into the mechanisms that filter species presence and abundance within a community. Trait coordination is expected be especially important for community assembly along environmental gradients, yet how trait coordination affects species presence versus abundance is incompletely understood. Our results suggest there are two levels of environmental filtering influencing understory herb community assembly in the southern Appalachian Mountains. Trait presence–absence, described by unweighted trait averages, showed weak environmental filtering, characterized by weak associations with environmental variables, context‐dependent trait coordination, and high dispersion within communities. By contrast, trait abundance, described by weighted trait averages, showed strong environmental filtering, with high convergence and consistent coordination among traits, especially in resource‐limited environments such as those found on southern aspects. These observed differences indicate that the constraints on trait abundance are stronger than the constraints on trait presence across resource gradients. Overall, we demonstrate that environmental filters influence species abundance by acting on multiple traits simultaneously, whereas species presence is determined by site‐level environmental conditions.

### 4.1 The effect of environmental gradients on single weighted and unweighted traits

Resource‐limited environments such as those found on south‐facing slopes were dominated by trait values associated with conservative plant strategies (e.g., smaller leaves and low stature) among both weighted and unweighted trait averages. However, environmental variables were stronger predictors of weighted than unweighted trait distributions. Taken together, these results support our hypothesis that environmental filtering on species abundance is stronger than filtering of species presence–absence in a community (H1). Weighted trait values of LA, LDMC, and SLA responded most strongly to soil moisture and soil N availability. As soil moisture decreased, we saw reductions in the weighted averages of SLA. Similarly, as NH_4_‐N availability decreased, so did the weighted averages of LA. Soil moisture plays a considerable role in controlling the availability of soil resources like NH_4_‐N (Leuschner & Lendzion, 2009), which in turn places limits on the abundance and distribution of understory herbs (Whittaker, 1956). The leaf patterns we observed are consistent with the expected physiological trade‐offs mediated by N availability and soil moisture (Tilman, 1990; Westoby, 1998) and suggest that filtering of trait diversity has resulted in environmentally mediated fitness differences among species with different functional strategies (Chave et al., 2009; Wright et al., 2004). This is not surprising as both LA and SLA are associated with plant carbon economy and relative growth rate. An increase in the weighted averages of these traits thus indicates that conditions of high resource availability favor species that can maximize carbon gain (Gaudet & Keddy, 1988; Westoby, 1998; Wright et al., 2007).

Surprisingly, weighted averages for LDMC deviated from the predictions of the leaf economics spectrum (Wright et al., 2004) in that LDMC increased as soil moisture increased. Also, unweighted and weighted δ^13^C were more depleted in the warmest and driest plots, which indicates that plants were not adjusting their water use efficiency in drier plots such as those located at low elevation, on south‐facing slopes. One explanation for the discrepancy between our data and theoretical expectations may be that environmental constraints often do not exert selection on single traits but instead exert selection on multiple traits simultaneously (Muscarella & Uriarte, 2016). Thus, species with suboptimal values in individual traits may persist in an environment but never reach high abundance there (Cingolani et al., 2007; Keddy, 1992).

### 4.2 Dispersion of unweighted and weighted traits across the resource gradients

We also observed lower CWV of weighted H and SLA as soil moisture and N concentration declined, which is in line with previous studies of herb community assembly (Bernard‐Verdier et al., 2012; Freschet et al., 2011; Violle et al., 2012). These results offer additional support for strong environmental filtering in constraining local trait diversity under limiting conditions for plant growth and support our hypothesis that species abundance is strongly determined by the magnitude of trait–environment correspondence (H2). Numerous investigations have demonstrated resource‐limited environments are more restrictive on the variation and distribution of plant functional traits (Díaz et al., 1998; Laliberté et al., 2014; Lebrija‐Trejos et al., 2010; Weiher & Keddy, 1995). Alternatively, high resource availability resulted in higher CWV of weighted H and SLA, which demonstrates that filtering does not always result in the exclusion of species with different trait values but rather alters their chances of reaching high abundance (Grime, 1998; Whittaker, 1965). SEMs specified on unweighted trait distributions similarly demonstrated weak but direct responses to environmental variation (Figure 5a,c, Appendices S9 and S11), which coupled with high variance among unweighted trait values, adds additional support for weaker filtering on species presence than abundance within a community.

### 4.3 The importance of trait coordination in explaining unweighted and weighted trait distributions

The importance of trait coordination in structuring weighted functional trait distributions supports the idea that trait coordination is tightly correlated with fitness and performance and therefore exerts a strong influence on species abundance (H3) (Laughlin & Messier, 2015; Muscarella & Uriarte, 2016). To date, most investigations have explored variation in trait coordination at regional or larger scales (Díaz et al., 2016; Reich et al., 1997; Wright et al., 2004), leaving considerable gaps in our understanding of how trait coordination may influence trait distributions along local environmental gradients. We found that coordination among weighted traits was uniform across the resource gradients (Figure 3b,d) and that soil and microclimate variables acted on weighted trait distributions indirectly via their influence on trait coordination (Figure 5b,d, Appendices S9 and S11). These results indicate that species abundance in a community is strongly determined by coordination among multiple traits related to different ecological strategies such as resource acquisition and carbon economy (sensu Ackerly et al., 2002).

Alternatively, coordination among unweighted trait averages was less consistent, depending strongly on environmental context. For instance, coordination among unweighted values of community‐average SLA and δ^13^C was particularly strong on water‐limited, low‐elevation, south‐facing slopes and is indicative of plants with water conservation strategies (Cernusak et al., 2009; Farquhar et al., 2002). We also found coordination between unweighted SLA and LN on less limiting high elevation north‐facing slopes, which likely stems from high N deposition rates at high elevations in the southern Appalachian Mountains (Block et al., 2012; Swank & Vose, 1997; Weathers et al., 2000). Indeed, our SEMs demonstrated that coordination among traits was not a significant factor in explaining unweighted trait distributions whereas weak direct relationships between unweighted trait distributions environmental variables were significant (Figure 5a,c, Appendices S9 and S11). Taken together, these results provide further evidence of the context‐dependent relationships between unweighted trait distributions and environmental variables in explaining trait presence within a community.

The observed differences in coordination among unweighted and weighted traits are consistent with patterns found at larger scales among a variety of habitats from temperate and tropical forests (Laughlin & Messier, 2015; Liu et al., 2012) to dry scrublands (Dwyer & Laughlin, 2017a) and suggest that, at local scales, the second‐level filter is a strong predictor of abundance but not necessarily presence (Ackerly et al., 2002; Cingolani et al., 2007). The importance of trait coordination in determining weighted trait distributions further suggests that evolutionary processes and resulting trade‐offs are shaping understory herb communities so that each trait, in concert with other traits, contributes to the community assembly process (Goud & Sparks, 2018; Read et al., 2014; Wiens et al., 2010). Overall, our results suggest the presence of two levels of environmental filters acting on understory herbs in southern Appalachian forests: the first level filter determines whether a species is present or absent across resource gradients whereas the second‐level filter, which acts on the degree of trait coordination, determines which species reach high abundance along the resource gradient (Cingolani et al., 2007; Leishman et al., 2010; Lodge, 1993; Westoby et al., 2002). Although additional research is needed to quantify biotic interactions to determine to what extent competition (i.e., limiting similarity) is also shaping functional trait distributions in this system, our study supports the view that environmental filters generally operate on multiple traits simultaneously rather than single traits in isolation (Kichenin et al., 2013; Read et al., 2014; Sundqvist et al., 2011).

Notably, the relationships between unweighted average traits and environmental variables left much of the variation in trait distributions unexplained. This may partly be due to the fact that only a snapshot of the local conditions experienced at each plot were measured. Lack of interannual variability in environmental variables at the plot level coupled with unknown dispersal history and stochastic effects may have also played a large role in determining the trait distributions we observed at both sites, which is especially important when considering presence and absence. Additionally, we did not measure other key environmental variables such as nitrogen supply rate, midsummer water potential, and temperature variability throughout the entire year. Environmental heterogeneity may be equally as important as environmental mean conditions in governing community assembly (Stark et al., 2017).

### 4.4 Implications for functional trait investigations

In conclusion, we show the importance of considering local environmental conditions and trait coordination when investigating understory herb community assembly. Our parallel assessment of single unweighted and weighted trait values provided novel insights into the importance of trait coordination in explaining understory herb species abundance in montane environments. Whereas niche differentiation governs trait distributions where resource availability is high (Kraft et al., 2008), strong environmental filtering appears to be governing trait distributions in resource‐limited settings. Stronger abiotic filters also manifest in stronger trait–environment relationships and thus environmental variables have greater predictive power in resource‐limited environments compared to high resource environments, especially for species abundances. However, our trait coordination results suggest evolutionary processes and associated trade‐offs are important components of the community assembly process as well. Because the relative importance of trait coordination is governed by environmental context and is subject to change under future climate scenarios, studying species‐specific fitness consequences of suites of traits in the context of abiotic variation has the potential to confer greater power to detect the complex patterns and underlying processes of community assembly for understory herbs.

## CONFLICT OF INTEREST

The authors declare no competing interests or conflicts of interest.

## AUTHOR CONTRIBUTION

**Matt Candeias:** Conceptualization (equal); Data curation (lead); Formal analysis (lead); Investigation (lead); Methodology (equal); Writing‐original draft (equal); Writing‐review & editing (equal). **Jennifer Fraterrigo:** Conceptualization (equal); Data curation (supporting); Formal analysis (supporting); Funding acquisition (lead); Methodology (supporting); Project administration (lead); Resources (lead); Supervision (lead); Validation (equal); Visualization (equal); Writing‐original draft (equal); Writing‐review & editing (equal).

## Supporting information

Appendix S1‐S11

## Data Availability

Coweeta Long Term Ecological Research Program & Fraterrigo, 2020. Trait coordination and environmental filters shape functional trait distributions of forest understory herbs ver 13. Environmental Data Initiative. https://doi.org/10.6073/pasta/2f2300df5d984581895216fcd96cff08
