# Supplementary material for: Trait coordination and environmental filters shape functional trait distributions of forest understory herbs
Source: Ecol Evol. 2020 Nov 26;10(24):14098–112. doi: 10.1002/ece3.7000 (PMC7771138; doi:10.1002/ece3.7000)
Supplement: Supplementary file 1 — Appendix S1‐S11 [file ECE3-10-14098-s001.docx]

Supporting information to the paper Trait coordination and environmental filters shape functional trait distributions of forest understory herbs. *Ecology and Evolution.*

**Appendix S1.** Information on traits measured including units, function, method of measurement, and relevant citations.

| **Trait** | **Unit** | **Function** |  | **Measurement** |  | **Reference** |
| --- | --- | --- | --- | --- | --- | --- |
| Height (H) | cm | Associated with various aspects of plant fitness including growth form, competitive vigor, and potential lifespan |  | Shortest distance on the main stem between the upper-most photosynthetic leaf and the ground level or upper-most portion of the rosette leaves. |  | Gaudet & Keddy 1988, Niklas 1994,  Westoby 1998 |
| Leaf Area (LA) | mm^2^ | Related to variation in climate, coping with heat, cold, and drought stress, and overall ecological strategy |  | Collected fully expanded, hardened leaves from adult plants, excluding the petiole. For species with compound leaves, we measured all leaflets without removing the rachis. Cut leaves were placed on a solid background accompanied by a 1 cm^2^ red square. Photographs were taken and uploaded into the automated digital image analysis program Easy Leaf Area (Easlon & Bloom 2014) |  | Ackerly *et al*. 2002, Givnish 1979,  Westoby *et al*. 2002 |
| Specific Leaf Area (SLA) | mm^-2^ mg^-1^ | Related to potential relative growth rate (RGR), leaf lifespan, photosynthetic rate, and coping with environmental stress |  | Collected fully expanded, hardened leaves from adult plants, excluding the petiole. Cut leaves were stored in plastic bags on ice until returning to the lab upon which they were weighed. After weighing, leaves were placed in paper envelopes and placed in a drying over at 70 ^o^C for 72 hours. We then weighed each leaf to determine its dry mass and calculated SLA by dividing the one-sided leaf area by its oven-dry mass. |  | Reich *et al*. 1992, Westoby *et al*. 2002, Wright *et al*. 2007 |

| **Trait** | **Unit** | **Function** |  | **Measurement** |  | **Reference** |
| --- | --- | --- | --- | --- | --- | --- |
| Leaf Dry Matter Content (LDMC) | mg g^-1^ | Related to potential RGR, leaf lifespan, and overall ecosystem productivity |  | Collected fully expanded, hardened leaves from adult plants, excluding the petiole. Cut leaves were stored in plastic bags on ice until returning to the lab upon which they were weighed. After weighing, leaves were placed in paper envelopes and placed in a drying over at 70 ^o^C for 72 hours. We then weighed each leaf to determine its dry mass and calculated LDMC by dividing the oven-dry mass of each leaf by its water-saturated fresh mass. |  | Díaz *et al*. 2004,  Niinemets 2001,  Westoby 1998 |
| Leaf Nitrogen (LN) | mg N g^-1^ | Indicator of vegetation composition, functioning, and nutrient limitation at the community level, and photosynthetic rate |  | Pulverized each leaf sample into a fine powder. Samples were then analyzed for total C and N, and δ^13^C on a Costech 4010 CHNSO Elemental Analyzer (Costech Analytical Technologies Inc., Valencia, CA, USA) interfaced with an isotope ratio mass spectrometer (Thermo Fisher Delta V Advantage, Fisher Scientific) at the University of Illinois. |  | Koerselman & Meuleman 1996,  Pérez-Harguindeguy *et al*. 2016 |
| Leaf δ^13^C (δ^13^C) | ‰ | Indication of water use efficiency |  | Pulverized each leaf sample into a fine powder. Samples were then analyzed for total C and N, and δ^13^C on a Costech 4010 CHNSO Elemental Analyzer (Costech Analytical Technologies Inc., Valencia, CA, USA) interfaced with an isotope ratio mass spectrometer (Thermo Fisher Delta V Advantage, Fisher Scientific) at the University of Illinois. |  | Cernusak *et al*. 2009,  Farquhar *et al*. 1989 |

Supporting information to the paper Trait coordination and environmental filters shape functional trait distributions of forest understory herbs. *Ecology and Evolution.*

**Appendix S2.** List of species by elevation and aspect found at both study sites (Coweeta Hydrologic Lab and Mainspring Land Trust). List represents species observations, not total number of each species at each site. All identification is done at the species level where possible.

| **High North** | **High South** | **Low North** | **Low South** |
| --- | --- | --- | --- |
| *Actaea pachypoda* | *Adiantum pedatum* | *Actaea pachypoda* | *Actaea pachypoda* |
| *Adiantum pedatum* | *Amphicarapaea bracteata* | *Adiantum pedatum* | *Arisaema triphyllum* |
| *Amphicarapaea bracteata* | *Arisaema triphyllum* | *Amphicarapaea bracteata* | *Botrypus virginianus* |
| *Angelica* sp | *Aristolochia macrophylla* | *Arisaema triphyllum* | *Chimaphila maculata* |
| *Arisaema triphyllum* | *Athyrium* sp | *Aristolochia macrophylla* | *Desmodium nudiflorum* |
| *Aristolochia macrophylla* | *Botrypus virginianus* | *Boehmeria cylindrica* | *Desmodium paniculatum* |
| *Athyrium filix-femina* | *Bristly Sunflower* | *Botrypus virginianus* | *Erigeron pulchellus* |
| *Athyrium* sp | *Campanula americana* | *Carex pensylvanica* | *Euonymous* sp |
| *Boehmeria cylindrica* | *Chimaphila maculata* | *Chimaphila maculata* | *Eurybia divaricata* |
| *Boehmeria* sp | *Circaea lutetiana* | *Cimicifuga racemosa* | *Galax urceolata* |
| *Botrychium multifidum* | *Conopholis americana* | *Circaea lutetiana* | *Galearis spectabilis* |
| *Botrypus virginianus* | *Dasistoma macrophylla* | *Clintonia umbellulata* | *Gallium* sp |
| *Cardamine diphylla* | *Desmodium nudiflorum* | *Conopholis americana* | *Goodyera pubescens* |
| *Caulophyllum thalictroides* | *Dichanthelium clandestinum* | *Cypripedium parviflorum* | *Mediola virginica* |
| *Circaea lutetiana* | *Dichanthelium* sp | *Dasistoma macrophylla* | *Melampyrum lineare* |
| *Conopholis americana* | *Dioscorea villosa* | *Desmodium nudiflorum* | *Mianthemum racemosum* |
| *Dasistoma macrophylla* | *Dryopteris marginalis* | *Dioscorea villosa* | *Monotropa hypopitys* |
| *Deparia acrostichoides* | *Euonymous* sp | *Enemion biternatum* | *Panax quinquefolius* |
| *Dioscorea villosa* | *Eurybia divericata* | *Erigeron pulchellus* | *Partheocissus vitacea* |
| *Dryopteris marginalis* | *Galearis spectabilis* | *Euonymous* sp | *Phryma leptostachya* |
| *Dryopteris* sp | *Gallium lanceolatum* | *Eurybia divericata* | *Polystichum acrostichoides* |
| *Eurybia divericata* | *Gallium* sp | *Galearis spectabilis* | *Prenanthes* sp |
| *Galearis spectabilis* | *Goodyera pubescens* | *Gallium lanceolatum* | *Prenanthes trifoliata* |
| *Gallium lanceolatum* | *Helianthus* sp | *Gallium* sp | *Pteridium aquilinum* |
| *Gallium* sp | *Laportea canadensis* | *Helianthus* sp | *Ranunculus* sp |
| *Geum* sp | *Lilium superbum* | *Houstonia* sp | *Rubus* sp |
| *Goodyera pubescens* | *Lysimachia quadrifolia* | *Mediola virginica* | *Sanicula gregaria* |
| *Impatiens* sp | *Lysimachia* sp | *Melampyrum pratense* | *Smilax* sp |
| *Lysimachia quadrifolia* | *Mediola virginica* | *Mianthemum racemosum* | *Symphyotrichum* sp |
| *Mediola virginica* | *Mianthemum racemosum* | *Mitchella repens* | *Tipularia discolor* |
| *Melampyrum pratense* | *Monotropa uniflora* | *Monarda* sp | *Toxicodendron radicans* |
| *Mianthemum racemosum* | *Oxalis grandis* | *Osmunda cinnamomea* | *Triphora trianthophoros* |
| *Monarda* sp | *Packera obovata* | *Panax quinquefolius* | *Uvularia perfoliata* |
| *Osmorhiza claytonii* | *Panax quinquefolius* | *Partheocissus vitacea* | *Uvularia sessilifolia* |
| *Osmorhiza* sp | *Panicum* sp | *Phegopteris connectilis* | *Viola canadensis* |
| *Osmunda cinnamomea* | *Partheocissus vitacea* | *Phryma leptostachya* | *Viola hastata* |
| *Osmunda claytoniana* | *Passiflora lutea* | *Polystichum acrostichoides* | *Viola palmata* |
| *Partheocissus vitacea* | *Phryma leptostachya* | *Potentilla* sp | *Viola pensylvanica* |
| *Pedicularis canadensis* | *Polystichum acrostichoides* | *Prenanthes trifoliata* | *Viola* sp |
| *Phryma leptostachya* | *Potentilla* sp | *Prosartes languinosa* |  |
| *Pilea pumila* | *Prenanthes trifoliata* | *Sanguinaria canadensis* |  |
| *Podophyllum peltatum* | *Prosartes languinosa* | *Sanicula gregaria* |  |
| *Polygonum* sp | *Pycnanthemum* sp | *Scutellaria* sp |  |
| *Polystichum acrostichoides* | *Rubus* sp | *Smilax* sp |  |
| *Prenanthes trifoliata* | *Sanguinaria canadensis* | *Symphyotrichum* sp |  |
| *Prosartes languinosa* | *Sanicula gregaria* | *Thalictrum dioicum* |  |
| *Pycnanthemum* sp | *Smilax herbacea* | *Thelypteris noveboracensis* |  |
| *Rubus* sp | *Smilax* sp | *Tierella cordifolia* |  |
| *Sanguinaria canadensis* | *Symphyotrichum* sp | *Toxicodendron radicans* |  |
| *Sanicula gregaria* | *Thalictrum dioicum* | *Trillium* sp |  |
| *Smilax herbacea* | *Tradescantia virginiana* | *Uvularia sessilifolia* |  |
| *Smilax* sp | *Uvularia perfoliata* | *Veratrum parviflorum* |  |
| *Stachys latidens* | *Uvularia sessilifolia* | *Viola blanda* |  |
| *Symphyotrichum* sp | *Veratrum parviflorum* | *Viola canadensis* |  |
| *Thalictrum dioicum* | *Viola canadensis* | *Viola hastata* |  |
| *Thelypteris noveboracensis* | *Viola hastata* | *Viola palmata* |  |
| *Tierella cordifolia* | *Viola palmata* | *Viola pensylvanica* |  |
| *Toxicodendron radicans* | *Viola pensylvanica* |  |  |
| *Uvularia perfoliata* | *Vitis* sp |  |  |
| *Uvularia sessilifolia* |  |  |  |
| *Veratrum parviflorum* |  |  |  |
| *Viola blanda* |  |  |  |
| *Viola canadensis* |  |  |  |
| *Viola hastata* |  |  |  |
| *Viola pensylvanica* |  |  |  |
| *Viola pubescens* |  |  |  |

Supporting information to the paper Trait coordination and environmental filters shape functional trait distributions of forest understory herbs. *Ecology and Evolution.*

**Appendix S3.** Principal component analysis (PCA) loadings. PCA identified two significant axes (eigenvalues > 1) of the variation in resource conditions.

| **Variable** | **PC1** | **PC2** |
| --- | --- | --- |
| **Soil Moisture** | -0.40 | -0.16 |
| **Average Temperature** | 0.52 | -0.25 |
| **Maximum Temperature** | 0.45 | 0.11 |
| **Minimum Temperature** | 0.40 | -0.40 |
| **pH** | 0.14 | -0.08 |
| **NO_3_-N** | -0.17 | 0.09 |
| **NH_4_-N** | -0.07 | -0.57 |
| **PO_4_** | 0.13 | -0.17 |
| **Soil N Concentration** | -0.34 | -0.45 |
| **PAR** | -0.08 | -0.40 |
|  |  |  |
| **Percent Total Variation Explained** | 29.4% | 19.7% |

Supporting information to the paper Trait coordination and environmental filters shape functional trait distributions of forest understory herbs. *Ecology and Evolution.*


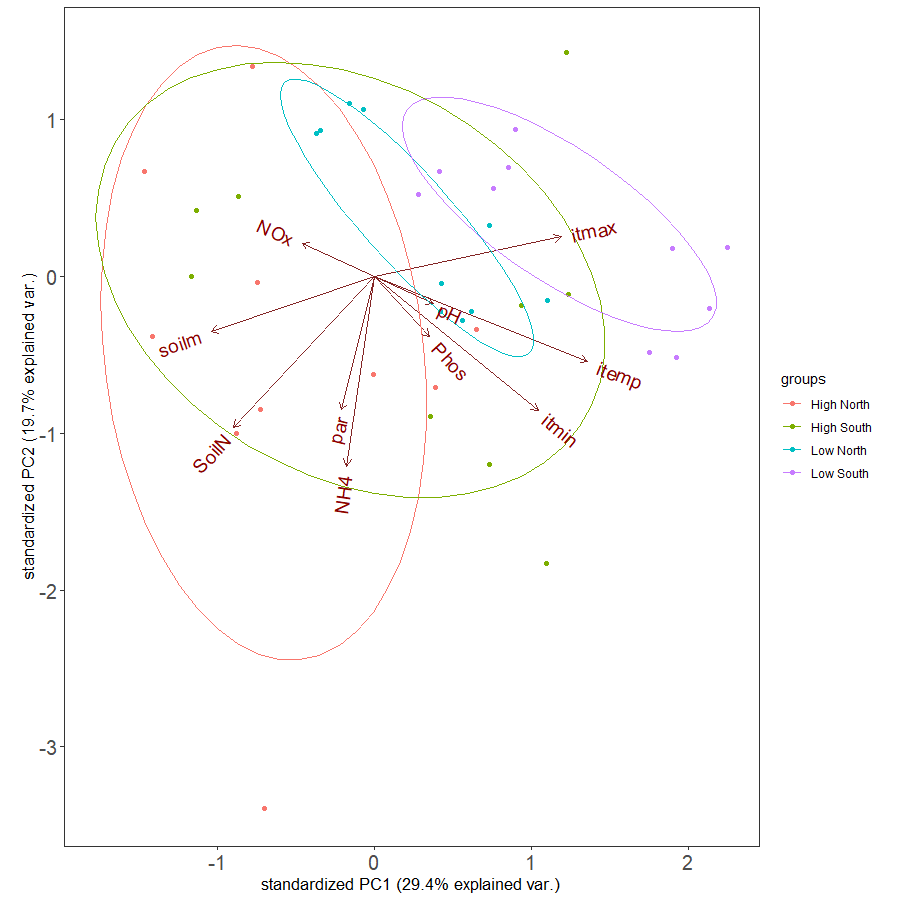


**Appendix S4.** Principal component analysis (PCA) of 10 environmental variables that are associated with plots (colored circles) and topographic position (ellipses). Abbreviations are as follows: maximum temperature, itmax; soil pH, pH; average temperature, itemp; minimum temperature, itmin; PO4, Phos; NH4-N, NH4; PAR; Soil N Concentration, SoilN; soil moisture, soilm; and NO3-N, NOx. PCA axis 1 corresponds to soil temperature, which increases from left to right and soil moisture, which decreases from left to right. Axis 1 explained 29.4% of the total variance extracted from our dataset and axis 2 explained 19.7%

Supporting information to the paper Trait coordination and environmental filters shape functional trait distributions of forest understory herbs. *Ecology and Evolution.*

**Appendix S5.** Unweighted and weighted trait means (+ SE) by topographic position averaged across sites. Abbreviations are as follows: specific leaf area, SLA; leaf dry matter content, LDMC; leaf nitrogen, Leaf N.

| **Elevation & Aspect** | | | | |
| --- | --- | --- | --- | --- |
| **Trait** | **High North** | **High South** | **Low North** | **Low South** |
| **Unweighted Height (cm)** | 31.1 + 5.49 | 25.1 + 6.06 | 15.4 + 5.92 | 11.1 + 6.49 |
| **Weighted Height (cm)** | 397.9 + 58.4 | 92.4 + 66.8 | 265.1 + 64.9 | 50.2 + 73.4 |
| **Unweighted Leaf Area (mm^2^)** | 106.8 + 15.0 | 38.2 + 17.7 | 76.8 + 17.2 | 29.9 + 20.0 |
| **Weighted Leaf Area (mm^2^)** | 519.2 + 79.9 | 119.5 + 92.1 | 390.6 + 89.5 | 61.9 + 102.0 |
| **Unweighted SLA (mm^-2^ mg^-1^)** | 571.0 + 87.9 | 452.0 + 95.3 | 546.0 + 93.5 | 454.0 + 100.9 |
| **Weighted SLA (mm^-2^ mg^-1^)** | 761.0 + 167.0 | 250.0 + 177.0 | 639.0 + 174.0 | 138.0 + 184.0 |
| **Unweighted LDMC (mg g^-1^)** | 0.22 + 0.03 | 0.15 + 0.03 | 0.26 + 0.03 | 0.24 + 0.04 |
| **Weighted LDMC (mg g^-1^)** | 213.2 + 40.1 | 84.5 + 42.5 | 180.7 + 42.5 | 34.9 + 45.7 |
| **Unweighted Leaf N (mg N g^-1^)** | 2.27 + 0.11 | 2.15 + 0.13 | 1.84 + 0.13 | 1.90 + 0.15 |
| **Weighted Leaf N (mg N g^-1^)** | 94.8 + 14.7 | 25.4 + 16.4 | 62.9 + 16.0 | 14.0 + 17.8 |
| **Unweighted δ^13^C (‰)** | -30.4 + 0.78 | -30.1 + 0.81 | -30.9 + 0.81 | -31.2 + 0.84 |
| **Weighted δ^13^C (‰)** | -429.9 + 88.5 | -140.4 + 94.4 | -350.9 + 92.9 | -76.6 + 98.9 |

Supporting information to the paper Trait coordination and environmental filters shape functional trait distributions of forest understory herbs. *Ecology and Evolution.*


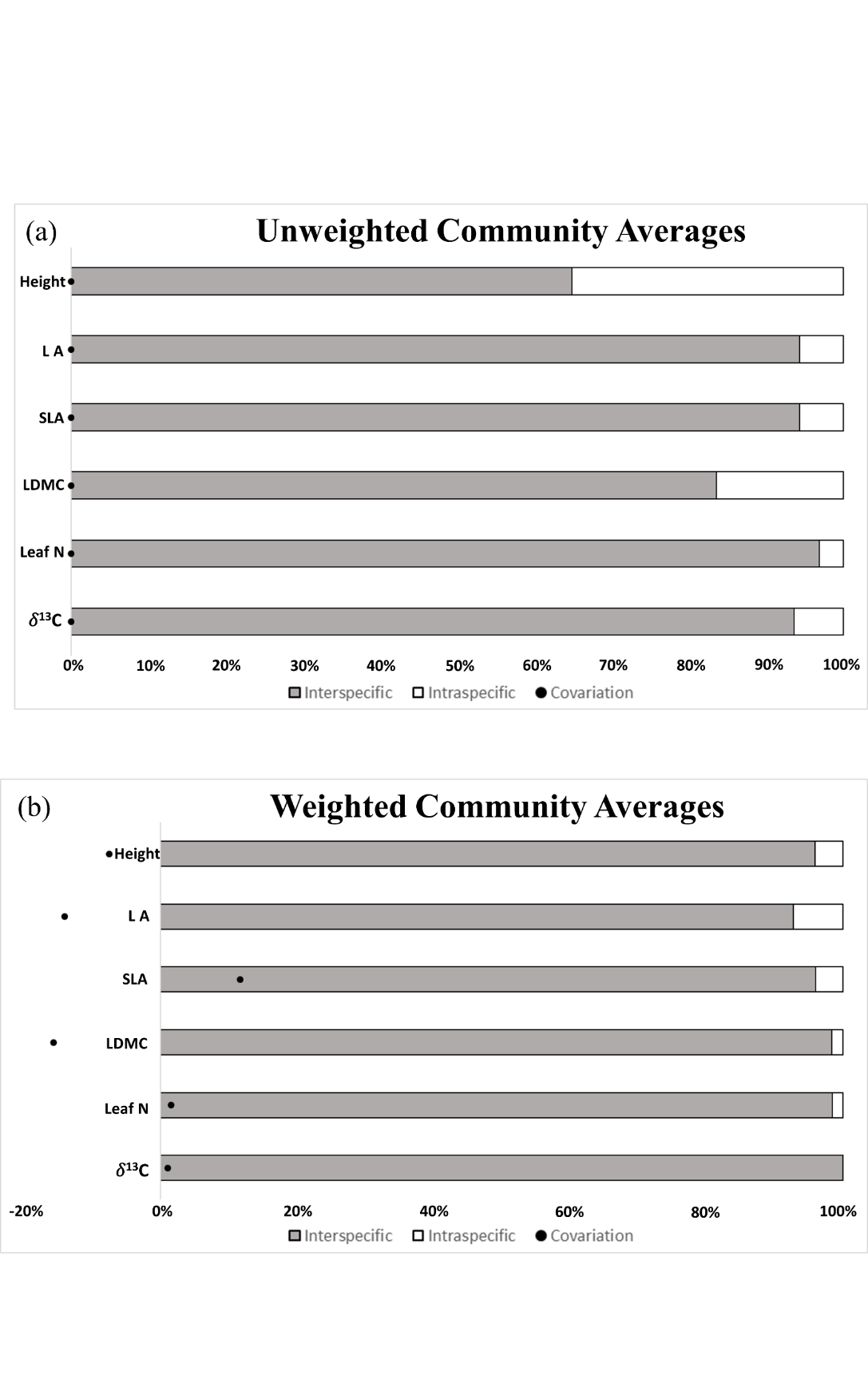


**Appendix S6.** Relative contribution of inter- (grey bars) and intraspecific (white bars) at both the unweighted (a) and weighted (b) level to total community means for the six traits measured. Covariation indicated by black squares. Covariation is represented by the difference between the total variance and the sum of the species turnover and intraspecific variation effects. Total variance greater than the sum of species turnover and intraspecific variation effects indicates positive covariance. Total variance less than the sum of species turnover and intraspecific variation effects indicates negative covariance. Negative covariation for all traits indicates that intraspecific variation and species turnover oppose each other (i.e., plots dominated by species with low trait values contain individuals with high trait values for their species). Abbreviations are as follows: leaf area, LA; specific leaf area, SLA; leaf dry matter content, LDMC; leaf nitrogen, Leaf N.

Supporting information to the paper Trait coordination and environmental filters shape functional trait distributions of forest understory herbs. *Ecology and Evolution.*

**Appendix S7**. Results of the linear mixed effect models on unweighted and weighted trait distributions. Significance indicated in grey. Abbreviations are as follows: specific leaf area, SLA; leaf dry matter content, LDMC; leaf nitrogen, Leaf N; maximum temperature, itmax; soil pH, pH; average temperature, Avg Temp; Min Temp, minimum temperature; photosynthetically active radiation, PAR; NH_4_, NH_4_-N, soil moisture, Soil M; and NO_3_-N, NOx.

| **Unweighted Weighted** | | | | | | | | | |
| --- | --- | --- | --- | --- | --- | --- | --- | --- | --- |
| **Trait** | **Variable** |  |  |  |  | **Variable** |  |  |  |
|  |  | **Beta** | **F_1,65_** | **P-Value** |  |  | **Beta** | **F_1,66_** | **P-Value** |
| **Height** | Elevation | 0.01 | 12.63 | 0.0007 |  | Elevation | -0.05 | 6.92 | 0.01 |
|  | Aspect | -0.01 | 0.01 | 0.92 |  | Aspect | -0.29 | 0.24 | 0.62 |
|  | Soil M | 0.67 | 12.45 | 0.0008 |  | Soil M | 11.74 | 25.58 | 0.0001 |
|  | Avg Temp | -4.15 | 1.91 | 0.17 |  | Avg Temp | -16.53 | 2.85 | 0.09 |
|  | PAR | -3.55 | 1.07 | 0.30 |  | NH_4_ | 213.04 | 7.88 | 0.0001 |
|  | NH_4_ | 4.11 | 0.32 | 0.56 |  | Soil N | 179.67 | 0.20 | 0.65 |
|  | Soil N | 16.03 | 0.01 | 0.90 |  | pH | 27.78 | 8.83 | 0.004 |
|  | pH | 1.77 | 0.36 | 0.54 |  | PO_4_ | 2.02 | 0.28 | 0.59 |
|  | PO_4_ | -7.14 | 0.11 | 0.73 |  |  |  |  |  |
|  |  |  |  |  |  |  |  |  |  |
|  |  | **Beta** | **F_1,66_** | **P-Value** |  |  | **Beta** | **F_1,66_** | **P-Value** |
| **Leaf Area** | Elevation | -0.002 | 0.42 | 0.51 |  | Elevation | -0.07 | 4.08 | 0.04 |
|  | Aspect | -0.06 | 1.25 | 0.26 |  | Aspect | -0.42 | 0.23 | 0.62 |
|  | Soil M | 4.44 | 5.31 | 0.02 |  | Soil M | 20.59 | 23.91 | 0.0001 |
|  | Avg Temp | -7.33 | 0.34 | 0.55 |  | Avg Temp | -37.73 | 1.70 | 0.19 |
|  | NH_4_ | -7.39 | 0.02 | 0.87 |  | NH_4_ | 332.93 | 7.88 | 0.006 |
|  | Soil N | 56.20 | 0.01 | 0.91 |  | pH | -1.86 | 8.41 | 0.005 |
|  | pH | 2.11 | 0.97 | 0.32 |  | PO_4_ | 12.88 | 0.18 | 0.66 |
|  | PO_4_ | -19.18 | 0.18 | 0.79 |  |  |  |  |  |
|  |  |  |  |  |  |  |  |  |  |

| **Unweighted Weighted** | | | | | | | | | |
| --- | --- | --- | --- | --- | --- | --- | --- | --- | --- |
| **Trait** | **Variable** |  |  |  |  | **Variable** |  |  |  |
|  |  | **Beta** | **F_1,66_** | **P-Value** |  |  | **Beta** | **F_1,62_** | **P-Value** |
| **SLA** | Elevation | -0.09 | 0.07 | 0.78 |  | Elevation | 2.95 | 6.02 | 0.01 |
|  | Aspect | 0.33 | 1.31 | 0.25 |  | Aspect | -0.56 | 0.10 | 0.74 |
|  | Soil M | 14.11 | 4.94 | 0.02 |  | Soil M | 295.52 | 31.57 | 0.0001 |
|  | Avg Temp | -15.27 | 0.03 | 0.85 |  | Avg Temp | 404.21 | 2.74 | 0.10 |
|  | NH_4_ | -6.22 | 0.0004 | 0.98 |  | Max Temp | -280.26 | 2.35 | 0.12 |
|  | Soil N | 261.49 | 0.60 | 0.44 |  | Min Temp | -222.76 | 0.59 | 0.44 |
|  | pH | 124.84 | 2.02 | 0.15 |  | PAR | 30.37 | 0.31 | 0.57 |
|  | PO_4_ | 76.11 | 0.44 | 0.51 |  | NH_4_ | 410.96 | 14.84 | 0.0003 |
|  |  |  |  |  |  | Soil N | 671.86 | 0.12 | 0.72 |
|  |  |  |  |  |  | pH | 44.22 | 10.56 | 0.001 |
|  |  |  |  |  |  | PO_4_ | -0.47 | 0.15 | 0.69 |
|  |  |  |  |  |  | Elevation x Aspect | -0.24 | 11.31 | 0.001 |
|  |  |  |  |  |  |  |  |  |  |
|  |  | **Beta** | **F_1,66_** | **P-Value** |  |  | **Beta** | **F_1,62_** | **P-Value** |
| **LDMC** | Elevation | -0.0001 | 3.15 | 0.08 |  | Elevation | 0.74 | 1.41 | 0.23 |
|  | Aspect | 0.0002 | 1.43 | 0.23 |  | Aspect | -0.15 | 0.01 | 0.89 |
|  | Soil M | -0.009 | 1.51 | 0.22 |  | Soil M | 79.35 | 15.56 | 0.0002 |
|  | Avg Temp | -0.01 | 0.38 | 0.53 |  | Avg Temp | 76.87 | 0.77 | 0.38 |
|  | NH_4_ | -0.03 | 0.51 | 0.47 |  | Max Temp | -64.61 | 0.91 | 0.34 |
|  | Soil N | 0.07 | 0.36 | 0.54 |  | Min Temp | -52.09 | 0.0003 | 0.98 |
|  | pH | -0.04 | 0.38 | 0.53 |  | PAR | 12.27 | 2.62 | 0.11 |
|  | PO_4_ | 0.08 | 1.55 | 0.21 |  | NH_4_ | 97.03 | 5.76 | 0.01 |
|  |  |  |  |  |  | Soil N | 165.13 | 0.15 | 0.07 |
|  |  |  |  |  |  | pH | 24.36 | 1.17 | 0.28 |
|  |  |  |  |  |  | PO_4_ | -11.43 | 0.03 | 0.85 |
|  |  |  |  |  |  | Elevation x Soil M | -0.06 | 9.07 | 0.003 |
|  |  |  |  |  |  |  |  |  |  |

| **Unweighted Weighted** | | | | | | | | | |
| --- | --- | --- | --- | --- | --- | --- | --- | --- | --- |
| **Trait** | **Variable** |  |  |  |  | **Variable** |  |  |  |
|  |  | **Beta** | **F_1,65_** | **P-Value** |  |  | **Beta** | **F_1,66_** | **P-Value** |
| **Leaf N** | Elevation | 0.001 | 9.13 | 0.003 |  | Elevation | -0.01 | 2.08 | 0.15 |
|  | Aspect | -0.0002 | 0.04 | 0.83 |  | Aspect | -0.05 | 0.10 | 0.75 |
|  | Soil M | -0.03 | 12.66 | 0.0007 |  | Soil M | 3.69 | 10.09 | 0.002 |
|  | Avg Temp | 0.006 | 0.02 | 0.87 |  | Avg Temp | -10.93 | 0.001 | 0.97 |
|  | NH_4_ | -0.21 | 0.59 | 0.44 |  | NH_4_ | 54.97 | 8.79 | 0.004 |
|  | Soil N | -6.61 | 0.05 | 0.81 |  | Soil N | 39.49 | 0.22 | 0.63 |
|  | pH | 0.49 | 6.42 | 0.01 |  | pH | 23.48 | 1.44 | 0.23 |
|  | PO_4_ | -0.05 | 0.86 | 0.35 |  | PO_4_ | 2.43 | 0.01 | 0.91 |
|  | Soil M x Soil N | 0.43 | 6.34 | 0.01 |  |  |  |  |  |
|  |  |  |  |  |  |  |  |  |  |
|  |  | **Beta** | **F_1,66_** | **P-Value** |  |  | **Beta** | **F_1,62_** | **P-Value** |
| **δ^13^C** | Elevation | 0.0001 | 2.49 | 0.11 |  | Elevation | 1.69 | 1.01 | 0.31 |
|  | Aspect | -0.001 | 0.36 | 0.54 |  | Aspect | -0.38 | 0.04 | 0.83 |
|  | Soil M | -0.15 | 0.32 | 0.56 |  | Soil M | 164.79 | 8.67 | 0.004 |
|  | Avg Temp | -0.65 | 10.17 | 0.002 |  | Avg Temp | 244.34 | 0.0001 | 0.99 |
|  | NH_4_ | 0.41 | 0.57 | 0.45 |  | Max Temp | -167.23 | 2.28 | 0.13 |
|  | Soil N | -0.08 | 0.006 | 0.93 |  | Min Temp | -123.44 | 0.15 | 0.69 |
|  | pH | -0.32 | 0.31 | 0.57 |  | PAR | 17.76 | 2.32 | 0.13 |
|  | PO_4_ | -0.19 | 0.06 | 0.80 |  | NH_4_ | 233.50 | 7.02 | 0.01 |
|  |  |  |  |  |  | Soil N | 324.75 | 0.12 | 0.72 |
|  |  |  |  |  |  | pH | 17.91 | 0.56 | 0.45 |
|  |  |  |  |  |  | PO_4_ | -6.19 | 0.0001 | 0.99 |
|  |  |  |  |  |  | Elevation x Soil M | -0.13 | 9.89 | 0.002 |
|  |  |  |  |  |  |  |  |  |  |

Supporting information to the paper Trait coordination and environmental filters shape functional trait distributions of forest understory herbs. *Ecology and Evolution.*

**
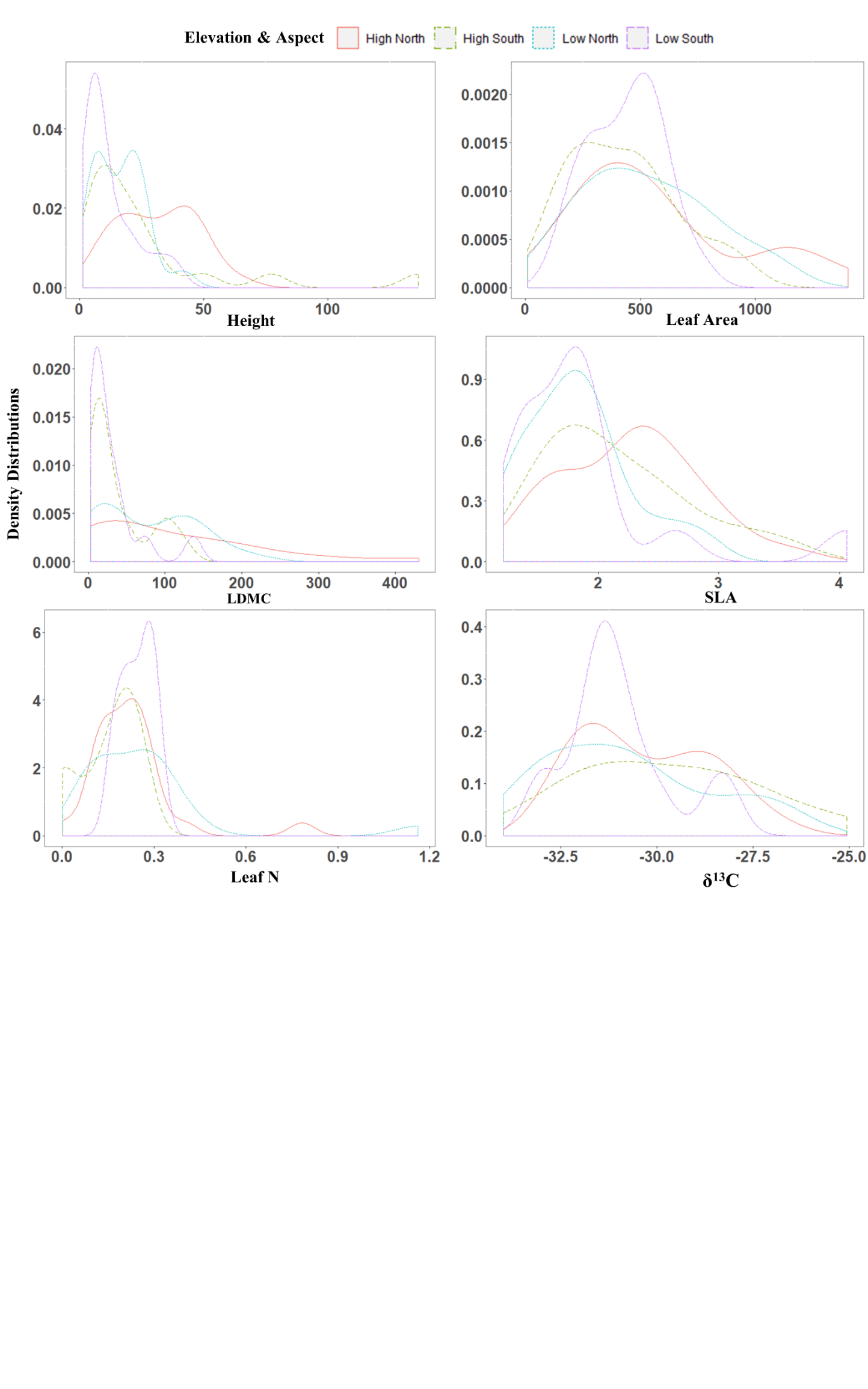
**

**Appendix S8.** Density distributions of six speices-level trait values for each elevation and aspect combination plotted using the ggridges package in R (R Development Core Team 2018). Topographic positions indicated by colored lines (High North; solid red, High South; dashed green, Low North; dashed blue, Low South; dashed purple). Despite plots having similar mean trait values, results of the Levene’s Test revealed traits exhibited more variation on north-facing slopes than on south-facing slopes, especially at lower elevations. Abbreviations are as follows: leaf area, LA; specific leaf area, SLA; leaf dry matter content, LDMC; leaf nitrogen, Leaf N.

Supporting information to the paper Trait coordination and environmental filters shape functional trait distributions of forest understory herbs. *Ecology and Evolution.*

**Appendix S9**. Structural equation model (SEM) estimates for unweighted and weighted height, Leaf Area (LA), Specific Leaf Area (SLA), Leaf Nitrogen (LN), and leaf δ^13^C. Arrows show direction of path from variables to the left to variables to the right of the arrow. Latent variables consist of: Edaphic; soil nutrients, LES; functional trait means, and Climate; moisture and temperature. Variable abbreviations are as follows: Leaf Economics Spectrum, LES; maximum temperature, max temp; soil pH, pH; average temperature, avg temp; minimum temperature, min temp; PO_4_, Phos; NH_4_-N, NH4; PAR; Soil N Concentration, SoilN; soil moisture, soilm; and NO_3_-N, NOx. Estimates are standardized coefficients, z is the test statistic, and p indicates level of significance. Variable Abbreviations are as follows: leaf area, LA; specific leaf area, SLA; leaf dry matter content, LDMC; leaf nitrogen, Leaf N; maximum temperature, max temp; soil pH, pH; average temperature, avg temp; minimum temperature, min temp; PO_4_, Phos; NH_4_-N, NH4; PAR; Soil N Concentration, SoilN; soil moisture, soilm; and NO_3_-N, NOx. For pathway interpretations see Appendix S3.

| **Trait Pathway Estimate *z p*** | | | | | | | | |
| --- | --- | --- | --- | --- | --- | --- | --- | --- |
| Unweighted Height |  | *Indirect effects* |  |  |  |  |  |  |
|  |  | NH_4_ → Edaphic |  | 0.12 |  | 1.89 |  | 0.05 |
|  |  | Soil N → Edaphic |  | 0.24 |  | 2.49 |  | 0.01 |
|  |  | LA → LES |  | 45.85 |  | 3.07 |  | 0.002 |
|  |  | δ^13^C → LES |  | 0.88 |  | 2.77 |  | 0.006 |
|  |  | Min Temp → Climate |  | 0.39 |  | 1.87 |  | 0.06 |
|  |  | Soil M → Climate |  | -2.85 |  | -2.59 |  | 0.006 |
|  |  |  |  |  |  |  |  |  |
|  |  | *Direct effects* |  |  |  |  |  |  |
|  |  | Edaphic → Height |  | 1.70 |  | 0.71 |  | 0.47 |
|  |  | LES → Height |  | 11.69 |  | 2.83 |  | 0.005 |
|  |  | Climate → Height |  | -2.66 |  | -0.78 |  | 0.43 |
|  |  |  |  |  |  |  |  |  |
| Weighted Height |  | *Indirect effects* |  |  |  |  |  |  |
|  |  | NH_4_ → Edaphic |  | 0.40 |  | 12.32 |  | 0.0001 |
|  |  | LA → LES |  | 398.80 |  | 12.32 |  | 0.0001 |
|  |  | Max Temp → Climate |  | 0.50 |  | 2.85 |  | 0.004 |
|  |  | Soil M → Climate |  | -2.33 |  | -3.07 |  | 0.002 |
|  |  |  |  |  |  |  |  |  |
|  |  | *Direct effects* |  |  |  |  |  |  |
|  |  | Edaphic → Height |  | 2.16 |  | 0.37 |  | 0.71 |
|  |  | LES → Height |  | 275.83 |  | 11.91 |  | 0.0001 |
|  |  | Climate → Height |  | -10.28 |  | -1.31 |  | 0.18 |
|  |  |  |  |  |  |  |  |  |

| **Trait Pathway Estimate *z p*** | | | | | | | | |
| --- | --- | --- | --- | --- | --- | --- | --- | --- |
| Unweighted LA |  | *Indirect effects* |  |  |  |  |  |  |
|  |  | NH_4_ → Edaphic |  | 0.10 |  | 1.63 |  | 0.10 |
|  |  | Soil N → Edaphic |  | 0.30 |  | 2.28 |  | 0.02 |
|  |  | Soil M → Climate |  | 1.72 |  | 5.39 |  | 0.0001 |
|  |  | Max Temp → Climate |  | -0.67 |  | -7.01 |  | 0.0001 |
|  |  | SLA → LES |  | 139.23 |  | 2.04 |  | 0.04 |
|  |  | LDMC → LES |  | 0.04 |  | 1.63 |  | 0.10 |
|  |  |  |  |  |  |  |  |  |
|  |  | *Direct effects* |  |  |  |  |  |  |
|  |  | Edaphic → LA |  | 0.60 |  | 0.06 |  | 0.23 |
|  |  | LES → LA |  | 62.33 |  | 1.20 |  | 0.22 |
|  |  | Climate → LA |  | 55.76 |  | 1.14 |  | 0.25 |
|  |  |  |  |  |  |  |  |  |
| Weighted LA |  | *Indirect effects* |  |  |  |  |  |  |
|  |  | Soil N → Edaphic |  | 0.17 |  | 12.32 |  | 0.0001 |
|  |  | SLA → LES |  | 520.23 |  | 12.32 |  | 0.0001 |
|  |  | Soil M → Climate |  | 1.94 |  | 6.04 |  | 0.0001 |
|  |  | Max Temp → Climate |  | -0.97 |  | -7.15 |  | 0.0001 |
|  |  |  |  |  |  |  |  |  |
|  |  | *Direct effects* |  |  |  |  |  |  |
|  |  | Edaphic → LA |  | 15.86 |  | 0.59 |  | 0.55 |
|  |  | LES → LA |  | 378.47 |  | 10.64 |  | 0.0001 |
|  |  | Climate → LA |  | -5.86 |  | -0.19 |  | 0.84 |
|  |  |  |  |  |  |  |  |  |

| **Trait Pathway Estimate *z p*** | | | | | | | | |
| --- | --- | --- | --- | --- | --- | --- | --- | --- |
| Unweighted SLA |  | *Indirect effects* |  |  |  |  |  |  |
|  |  | pH → Edaphic |  | 0.39 |  | 12.32 |  | 0.0001 |
|  |  | LDMC → LES |  | 0.15 |  | 12.32 |  | 0.0001 |
|  |  | Min Temp → Climate |  | 11.58 |  | 0.11 |  | 0.91 |
|  |  | Max Temp → Climate |  | 0.05 |  | 0.10 |  | 0.91 |
|  |  |  |  |  |  |  |  |  |
|  |  | *Direct effects* |  |  |  |  |  |  |
|  |  | Edaphic → SLA |  | 75.86 |  | 2.29 |  | 0.02 |
|  |  | LES → SLA |  | -37.10 |  | -1.18 |  | 0.23 |
|  |  | Climate → SLA |  | 7.67 |  | 0.11 |  | 0.91 |
|  |  |  |  |  |  |  |  |  |
| Weighted SLA |  | *Indirect effects* |  |  |  |  |  |  |
|  |  | Soil N → Edaphic |  | 0.17 |  | 12.32 |  | 0.0001 |
|  |  | δ^13^C → LES |  | 291.28 |  | 12.32 |  | 0.0001 |
|  |  | Soil M → Climate |  | -1.94 |  | -6.00 |  | 0.0001 |
|  |  | Max Temp → Climate |  | 0.98 |  | 7.14 |  | 0.0001 |
|  |  | Aspect → Climate |  | 6.30 |  | 0.41 |  | 0.67 |
|  |  |  |  |  |  |  |  |  |
|  |  | *Direct effects* |  |  |  |  |  |  |
|  |  | Edaphic → SLA |  | 4.33 |  | 0.46 |  | 0.64 |
|  |  | LES → SLA |  | 516.09 |  | 12.19 |  | 0.0001 |
|  |  | Climate → SLA |  | 0.74 |  | 0.07 |  | 0.94 |
|  |  |  |  |  |  |  |  |  |

| **Trait Pathway Estimate *z p*** | | | | | | | | |
| --- | --- | --- | --- | --- | --- | --- | --- | --- |
| Unweighted LN |  | *Indirect effects* |  |  |  |  |  |  |
|  |  | NH_4_ → Edaphic |  | 0.09 |  | 1.21 |  | 0.22 |
|  |  | Height → LES |  | 20.64 |  | 12.32 |  | 0.0001 |
|  |  | Avg Temp → Climate |  | 0.80 |  | 5.51 |  | 0.0001 |
|  |  | Max Temp → Climate |  | 1.12 |  | 7.93 |  | 0.0001 |
|  |  | Soil Moisture → Climate |  | -0.67 |  | -4.21 |  | 0.0001 |
|  |  | *Direct effects* |  |  |  |  |  |  |
|  |  | Edaphic → LN |  | 0.02 |  | 0.72 |  | 0.42 |
|  |  | LES → LN |  | 0.11 |  | 1.53 |  | 0.12 |
|  |  | Climate → LN |  | -0.15 |  | -1.97 |  | 0.04 |
|  |  |  |  |  |  |  |  |  |
| Weighted LN |  | *Indirect effects* |  |  |  |  |  |  |
|  |  | NH_4_ → Edaphic |  | 0.40 |  | 12.32 |  | 0.0001 |
|  |  | δ^13^C → LES |  | 291.28 |  | 12.32 |  | 0.0001 |
|  |  | Avg Temp → Climate |  | 1.25 |  | 12.32 |  | 0.0001 |
|  |  |  |  |  |  |  |  |  |
|  |  | *Direct effects* |  |  |  |  |  |  |
|  |  | Edaphic → LN |  | 2.31 |  | 0.80 |  | 0.42 |
|  |  | LES → LN |  | 59.51 |  | 10.60 |  | 0.0001 |
|  |  | Climate → LN |  | -6.63 |  | -2.42 |  | 0.01 |
|  |  |  |  |  |  |  |  |  |
|  |  |  |  |  |  |  |  |  |

| **Trait Pathway Estimate *z p*** | | | | | | | | | |  |
| --- | --- | --- | --- | --- | --- | --- | --- | --- | --- | --- |
| Unweighted δ^13^C |  | *Indirect effects* |  |  |  |  |  |  | | |
|  |  | NH_4_ → Edaphic |  | 0.10 |  | 1.45 |  | 0.14 | | |
|  |  | Soil N → Edaphic |  | 3.85 |  | 1.91 |  | 0.05 | | |
|  |  | LN → LES |  | 0.36 |  | 5.42 |  | 0.0001 | | |
|  |  | Soil M → Climate |  | 1.64 |  | 4.97 |  | 0.0001 | | |
|  |  | Max Temp → Climate |  | -0.71 |  | -6.99 |  | 0.0001 | | |
|  |  |  |  |  |  |  |  |  | | |
|  |  | *Direct effects* |  |  |  |  |  |  | | |
|  |  | Edaphic → δ^13^C |  | -0.03 |  | -0.19 |  | 0.84 | | |
|  |  | LES → δ^13^C |  | 2.15 |  | 5.40 |  | 0.0001 | | |
|  |  | Climate → δ^13^C |  | -1.13 |  | -2.48 |  | 0.01 | | |
|  |  |  |  |  |  |  |  |  | | |
| Weighted δ^13^C |  | *Indirect effects* |  |  |  |  |  |  | | |
|  |  | NH_4_ → Edaphic |  | 0.40 |  | 12.32 |  | 0.0001 | | |
|  |  | SLA → LES |  | 520.2 |  | 12.32 |  | | 0.0001 |  |
|  |  | Max Temp → Climate |  | -1.18 |  | -12.32 |  | 0.0001 | | |
|  |  |  |  |  |  |  |  |  | | |
|  |  | *Direct* |  |  |  |  |  |  | | |
|  |  | Edaphic → δ^13^C |  | 1.51 |  | 0.43 |  | 0.66 | | |
|  |  | LES → δ^13^C |  | 289.3 |  | 12.18 |  | 0.0001 | | |
|  |  | Climate → δ^13^C |  | 0.13 |  | 0.04 |  | 0.96 | | |
|  |  |  |  |  |  |  |  |  | | |
|  |  |  |  |  |  |  |  |  | | |

Supporting information to the paper Trait coordination and environmental filters shape functional trait distributions of forest understory herbs. *Ecology and Evolution.*

**
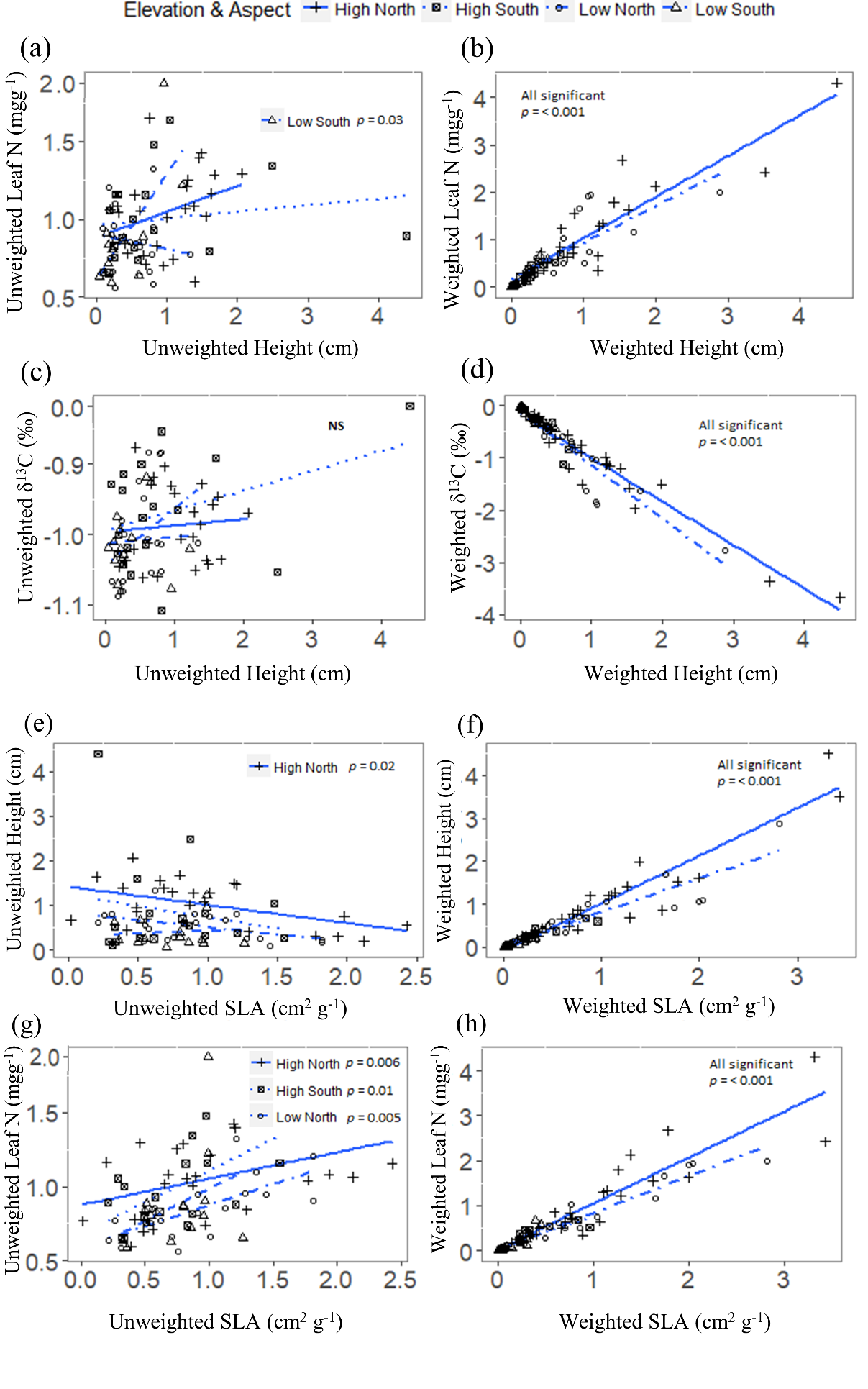
**

**Appendix S10.** Linear regression relationships between leaf and height trait averages based on topographic position. Covariation between unweighted height and leaf traits (a, c, e, g) is considerably less than coordination between leaf traits (b, d, f, h). Unweighted traits (a, c, e, g) show context-dependent coordination whereas weighted traits (b, d, f, h) show uniform coordination. Regression lines (blue) are shown for all elevation and aspect combinations. Significant relationships denoted by *p*-value within each plot. Axes reflect scaled and centered trait values to enable direct comparisons of effect sizes.

Supporting information to the paper Trait coordination and environmental filters shape functional trait distributions of forest understory herbs. *Ecology and Evolution.*

**
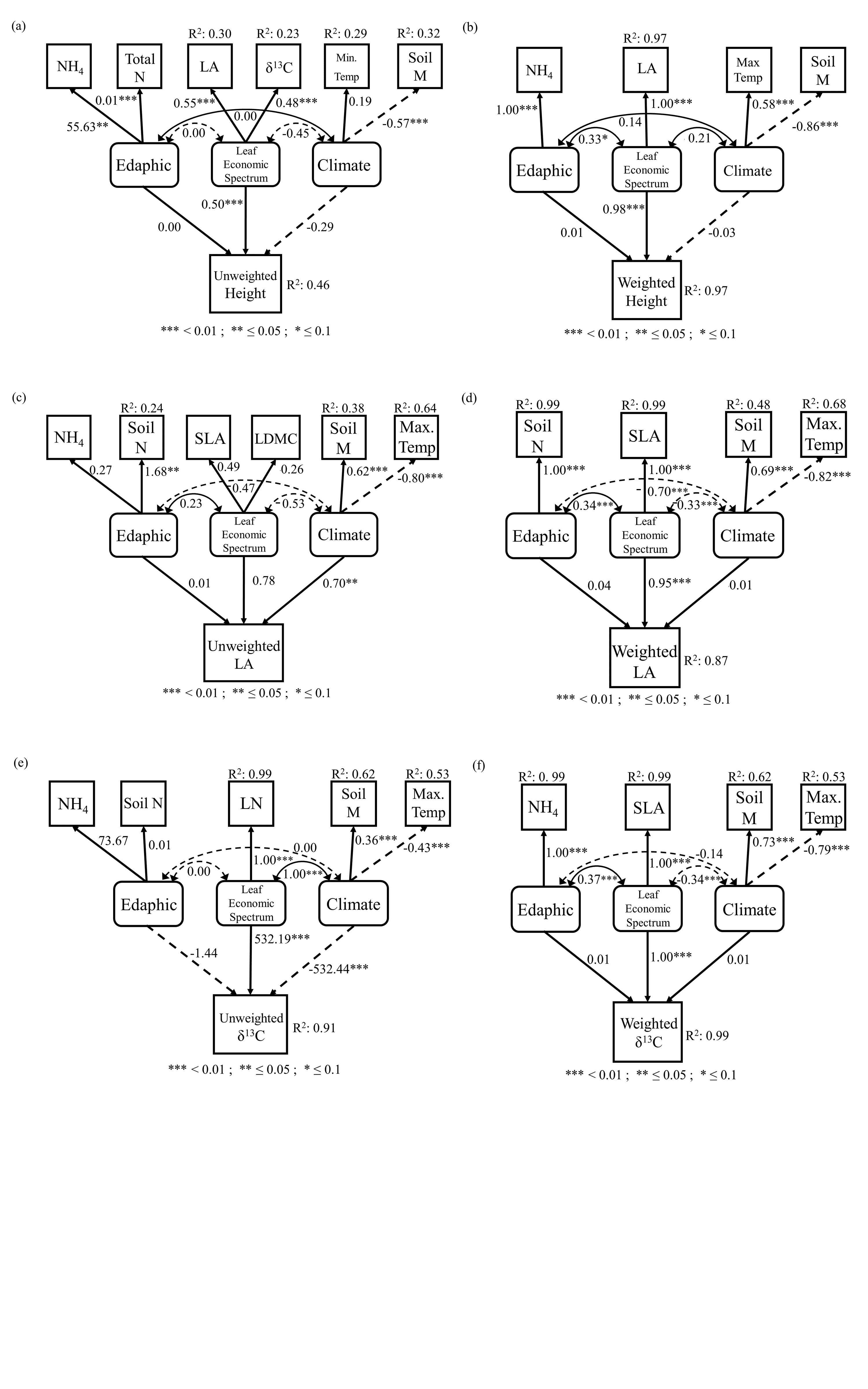
**

**Appendix S11.** Structural equation models fitted on both unweighted and weighted trait averages measured from understory herbs communities in the southern Appalachian Mountains H (a & b), LA (c & d), and δ^13^C (e & f). Measured variables are indicated by squares whereas latent variables are indicated by rounded rectangles. Solid arrows indicate positive relationships and dashed arrows indicate negative relationships. Significant paths are indicated by * (*** < 0.01; ** < 0.05; * < 0.1). Double headed arrows refer to covariance estimates. We fit models using the robust maximum likelihood method and overall goodness of fit estimated from chi-square statistic and the root mean square error of approximation (RMSEA). Variable abbreviations are as follows: Leaf area, LA; specific leaf area, SLA; leaf dry matter content, LDMC; leaf nitrogen, Leaf N; maximum temperature, max temp; soil pH, pH; average temperature, avg temp; minimum temperature, min temp; PO_4_, Phos; NH_4_-N, NH4; PAR; Soil N Concentration, SoilN; soil moisture, soilm; and NO_3_-N, NOx. We were unable to fit a model for LDMC at either level of trait aggregation and therefore omitted the SEM diagram.

**References**

Ackerly, D., Knight, C. A., Weiss, S. B., Barton, K., & Starmer, K. P. (2002). Leaf size, specific leaf area and microhabitat distribution of chaparral woody plants: Contrasting patterns in species level and community level analyses. *Oecologia*, *130*(3), 449–457. doi: 10.1007/s004420100805

Cernusak, L. A., Tcherkez, G., Keitel, C., Cornwell, W., Santiago, L. S., Knohl, A., *et al*. (2009). Why are non-photosynthetic tissues generally 13 C enriched compared with leaves in C 3 plants ? Review and synthesis of current hypotheses. *Functional Plant Biology*, (36), 199–213. doi: 10.1071/FP08216

Díaz, S., Hodgson, J. G., Thompson, K., Cabido, M., Cornelissen, J. H. C., Jalili, A., *et al*. (2004). The plant traits that drive ecosystems: evidence from three continents. *Journal of Vegetation Science*, *15*(3), 295–304. doi: 10.1111/j.1654-1103.2004.tb02266.x

Farquhar, G. D., Ehleringer, J. R., & Hubick, K. T. (1989). Carbon isotope discrimination and photosynthesis. Annual review of plant biology, 40(1), 503-537. doi: 10.1146/annurev.pp.40.060189.002443

Gaudet, C. L., & Keddy, P. A. (1988). A comparative approach to predicting competitive ability from plant traits. Nature, 334(6179), 242-243. doi: 10.1038/334242a0

Givnish, T. (1979). On the adaptive significance of leaf form. In Topics in plant population biology (pp. 375-407). Palgrave, London. doi: 10.1007/978-1-349-04627-0_17

Koerselman, W., & Meuleman, A. F. (1996). The vegetation N: P ratio: a new tool to detect the nature of nutrient limitation. Journal of applied Ecology, 1441-1450. doi: 10.2307/2404783

Niinemets, Ü. (2001). Global‐scale climatic controls of leaf dry mass per area, density, and thickness in trees and shrubs. *Ecology*, 82(2), 453-469. doi: 10.1890/0012-9658(2001)082[0453:GSCCOL]2.0.CO;2

Niklas, K. J. (1994). Plant allometry: the scaling of form and process. University of Chicago Press.

Pérez-Harguindeguy, N., Diaz, S., Gamier, E., Lavorel, S., Poorter, H., Jaureguiberry, P., *et al*. (2013). New handbook for stand-ardised measurement of plant functional traits worldwide. *Australian Journal of Botany* 61: 167-234. doi: 10.1071/BT12225_CO

Reich, P. B., Walters, M. B., & Ellsworth, D. S. (1992). Leaf life‐span in relation to leaf, plant, and stand characteristics among diverse ecosystems. Ecological monographs, 62(3), 365-392. doi: 10.2307/2937116

Westoby, M. (1998). A leaf-height-seed (LHS) plant ecology strategy scheme. *Plant and Soil*, *199*, 213–227. doi: 10.1023/A:1004327224729

Westoby, M., Falster, D. S., Moles, A. T., Vesk, P. A., & Wright, I. J. (2002). Plant ecological strategies: some leading dimensions of variation between species. Annual review of ecology and systematics, 33(1), 125-159. doi: 10.1146/annurev.ecolsys.33.010802.150452

Wright, I. J., Ackerly, D. D., Bongers, F., Harms, K. E., Ibarra-Manriquez, G., Martinez-Ramos, M., *et al*. (2007). Relationships among ecologically important dimensions of plant trait variation in seven neotropical forests. *Annals of Botany*, 99(5), 1003-1015. Doi: 10.1093/aob/mcl066
